# Opposite regulation of MDM2 and MDMX expression in acquisition of mesenchymal phenotype in benign and cancer cells

**DOI:** 10.18632/oncotarget.5392

**Published:** 2015-09-25

**Authors:** Eva Slabáková, Gvantsa Kharaishvili, Monika Smějová, Zuzana Pernicová, Tereza Suchánková, Ján Remšík, Stanislav Lerch, Nicol Straková, Jan Bouchal, Milan Král, Zoran Culig, Alois Kozubík, Karel Souček

**Affiliations:** ^1^ Department of Cytokinetics, Institute of Biophysics, Academy of Sciences of the Czech Republic, v.v.i., Brno, Czech Republic; ^2^ Center of Biomolecular and Cellular Engineering, International Clinical Research Center, St. Anne's University Hospital Brno, Brno, Czech Republic; ^3^ Department of Clinical and Molecular Pathology and Institute of Molecular and Translational Medicine, Faculty of Medicine and Dentistry, Palacky University, Olomouc, Czech Republic; ^4^ Department of Biochemistry, Faculty of Science, Masaryk University, Brno, Czech Republic; ^5^ Department of Experimental Biology, Faculty of Science, Masaryk University, Brno, Czech Republic; ^6^ Department of Urology, Faculty of Medicine and Dentistry, Palacky University, Olomouc, Czech Republic; ^7^ Division of Experimental Urology, Department of Urology, Medical University of Innsbruck, Austria

**Keywords:** epithelial-mesenchymal transition, MDM2/MDMX, SNAI2/SLUG, TWIST, prostate/breast cancer

## Abstract

Plasticity of cancer cells, manifested by transitions between epithelial and mesenchymal phenotypes, represents a challenging issue in the treatment of neoplasias. Both epithelial-mesenchymal transition (EMT) and mesenchymal-epithelial transition (MET) are implicated in the processes of metastasis formation and acquisition of stem cell-like properties. Mouse double minute (MDM) 2 and MDMX are important players in cancer progression, as they act as regulators of p53, but their function in EMT and metastasis may be contradictory. Here, we show that the EMT phenotype in multiple cellular models and in clinical prostate and breast cancer samples is associated with a decrease in MDM2 and increase in MDMX expression. Modulation of EMT-accompanying changes in MDM2 expression in benign and transformed prostate epithelial cells influences their migration capacity and sensitivity to docetaxel. Analysis of putative mechanisms of MDM2 expression control demonstrates that in the context of defective p53 function, MDM2 expression is regulated by EMT-inducing transcription factors Slug and Twist. These results provide an alternative context-specific role of MDM2 in EMT, cell migration, metastasis, and therapy resistance.

## INTRODUCTION

The metastatic process is a serious aspect of cancer disease that is tightly linked to cancer cell plasticity, epithelial-mesenchymal transition (EMT), mesenchymal-epithelial transition (MET), and acquisition of a stem-like cell phenotype, resulting in cancer cell dissemination and resistance to therapy [1]. EMT and MET are reversible processes that enable cell detachment from the primary tumor, penetration and survival in the circulation, and formation of metastasis at a distant site [2]. Both transitions are regulated by cooperation between multiple transcription factors and microRNAs whose functions may be overlapping, and effect of individual molecules in these processes can be time- and/or context-dependent [3]. We have previously shown that *SNAI2*/Slug is an early factor upregulated in transforming growth factor (TGF)-β-induced EMT of benign prostate epithelial cells, which is later accompanied by induction of zinc finger E-box binding homeobox (ZEB)1 and ZEB2 and deregulation of the miR-200 family of microRNAs [4]. Twist cooperation with Slug is necessary for EMT induction in mammary epithelial cells [5], while deregulation of miR-200c and miR-205 leads to EMT that is associated with clinically relevant insensitivity to docetaxel in prostate cells [6].

Stimuli that induce the EMT process include activation of signaling pathways by cytokines, oncogenes, or tumor microenvironment-associated molecules [3]. Importantly, EMT can also result from defects in a crucial tumor suppressor, p53. Both gain-of-function p53 mutations and p53 loss have been associated with EMT [7–9], while intact p53 function is important for maintenance of the epithelial phenotype [10, 11]. To ensure appropriate protection from DNA damage by inducing repair or cell death, p53 expression and activity is tightly regulated by mouse double minute homolog (MDM) 2 and its homolog MDMX. MDM2 controls p53 functions via several mechanisms, such as direct binding and occlusion of the transactivation domain, ubiquitination and degradation by the proteasome, and control of subcellular localization. MDMX lacks the E3 ubiquitin ligase activity of MDM2, but can regulate localization, stability, and function of both p53 and MDM2 [12, 13]. Inhibition of MDM2 was shown to sensitize various cancer cell lines to chemotherapy and the therapeutic strategy of p53 reactivation by MDM2 inhibitors and antagonists is being investigated in several clinical trials [14, 15].

Multiple p53-independent functions of MDM2 and MDMX were reported, which include effects on tumor cell proliferation, apoptosis, or invasion, in a background of p53 dysfunction [12, 16, 17]. In the context of clinical cancer metastasis, different outcomes of MDM2 expression were reported in various tissues. In colon cancer, MDM2 amplification or higher transcript abundance is associated with lower metastasis occurrence rates [18, 19], while in prostate and breast cancer, elevated MDM2 expression correlates with poor outcome [20, 21].

Different mechanisms by which MDM2 positively or negatively regulates EMT and metastasis have been proposed. On the one hand, MDM2 can promote cell motility and invasiveness by regulating E-cadherin degradation via its E3 ubiquitin ligase activity [22]. On the other hand, MDM2 can serve as an effector of wild-type (wt) p53 function in negative regulation of Slug, thereby preventing EMT and metastasis [11].

Our present study shows data supporting a context-dependent EMT-associated role of MDM2, in the regulation of cell migration. We show that in several cellular models and clinical tissue samples, the epithelial phenotype is associated with high expression of MDM2 and low expression of MDMX. Our data also show that EMT-inducing transcription factors Slug and Twist can regulate MDM2 expression, which, in turn, affects cell migration and sensitivity to chemotherapy.

## RESULTS

### EMT in prostate and breast cancer cell lines is associated with changes in MDM2 and MDMX expression pattern

**Figure 1:**
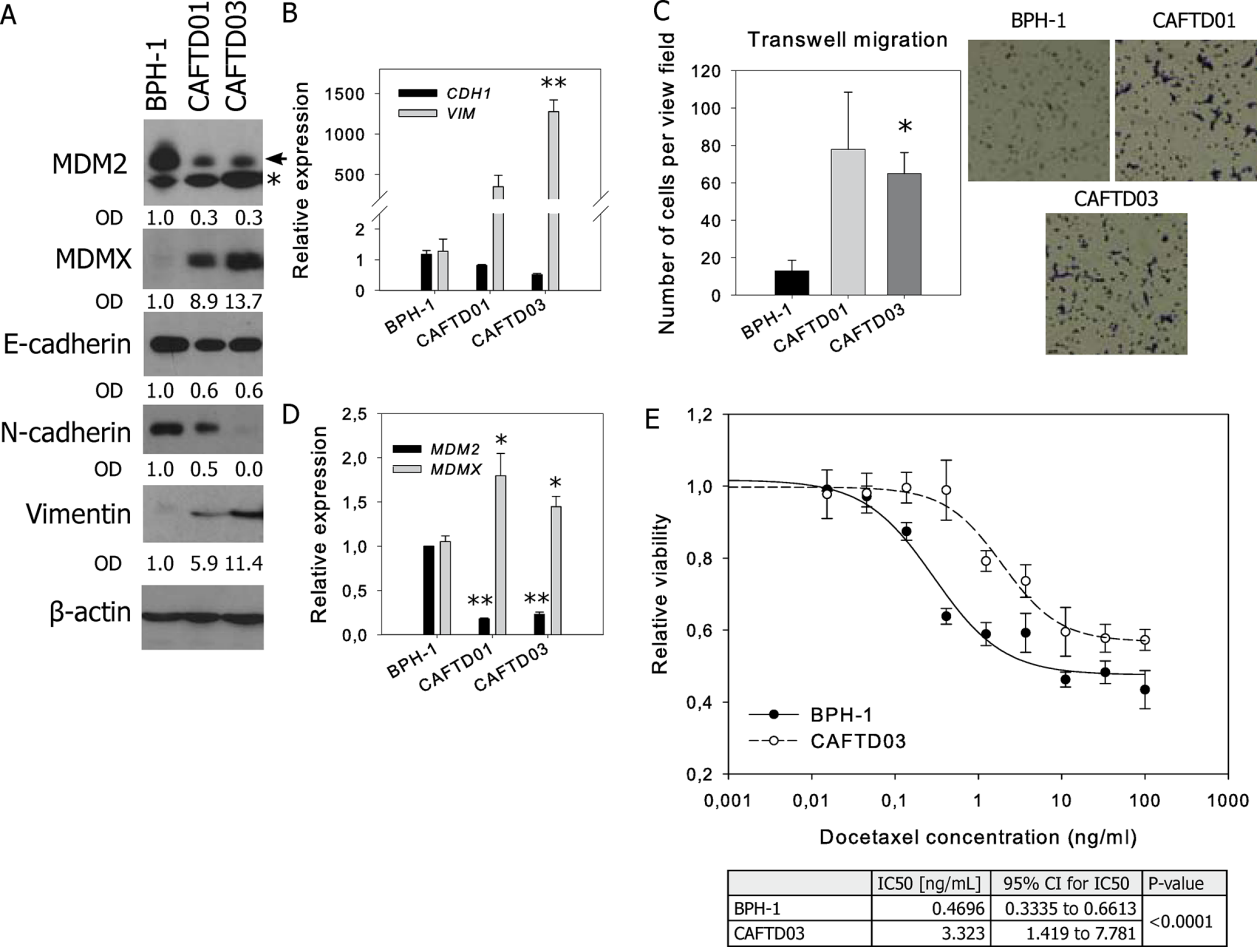
Tumorigenic prostate cell lines with mesenchymal characteristics and increased resistance to docetaxel are characterized by downregulation of MDM2 and upregulation of MDMX Western blotting and qRT-PCR analysis of MDM2, MDMX, epithelial and mesenchymal markers, and cell migration of human benign prostate BPH-1 cells and two independent tumorigenic clones, CAFTD01 and CAFTD03. **A.** Images of representative blots are shown; the full length MDM2 protein product is marked by an arrow, a faster-migrating product is marked by an asterisk (Supplementary Figure S1B). Relative protein expression was evaluated by measuring optical density (OD). **B–C.** PCR data represent mean ± SEM. (B) **P < 1.10^−3^ to BPH-1 cells, *n* = 3 (C) *P < 0.05, **P < 1.10^−6^ to BPH-1 cells, *n* = 7. **D.** Migration results through an uncoated 8 μm-pore transwell represent the average number (mean ± SD) of migrating cells in five independent viewing fields after 6 h of migration, from 3 independent experiments in technical duplicate; *P < 0.01 to BPH-1 cells. Photographs show results of a representative experiment at 40× magnification. **E.** Viability of BPH-1 and CAFTD03 cells treated with docetaxel for 72 h was analyzed using a luminescence-based ATP assay. Graphs show data from a representative experiment in technical triplicate. IC50 values were calculated from 3 independent experiments.

Multiple components of the tumor microenvironment, particularly factors secreted by stromal cells, activate cytokine- or oncogene-driven signaling pathways regulating cancer transformation and EMT. For instance, in immortalized BPH-1 cells derived from human benign prostate hyperplasia tissue [23], cancer transformation *in vivo* in the presence of cancer-associated fibroblasts gave rise to independent tumorigenic clones CAFTD01 and CAFTD03 with increased expression of markers of the mesenchymal phenotype [4, 24]. Our results show that the changes in the expression of epithelial (E-cadherin) and mesenchymal markers (vimentin, N-cadherin) are associated with enhanced migration potential (Figure 1A–1C, Supplementary Figure S1A). Notably, both CAFTD clones exhibiting the partial EMT phenotype showed decreased expression of MDM2 and increased expression of MDMX (Figure 1A, 1D, Supplementary Figure S1B–S1C). Besides the promotion of cellular migration, EMT is associated with increased resistance to chemotherapy [6]. We observed that compared to epithelial BPH-1 cells, tumorigenic CAFTD03 cells, whose phenotype is shifted towards mesenchymal cells, were less sensitive to docetaxel, a microtubule inhibitor used in standard chemotherapy of metastatic CaP (Figure 1E).

**Figure 2:**
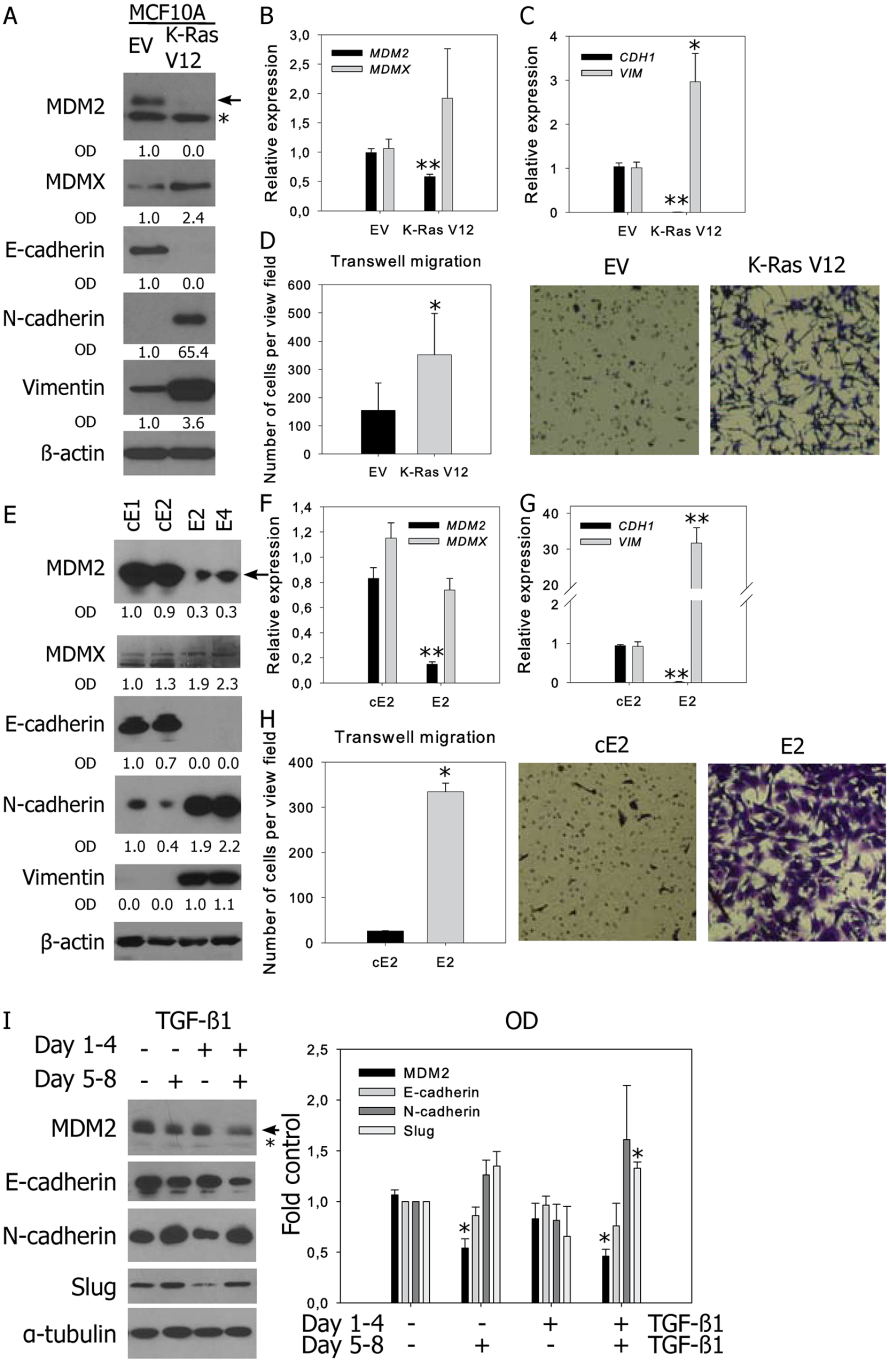
Decreased MDM2 expression is observed in prostate and breast cell lines with mesenchymal characteristics and in TGF-β-induced EMT Western blotting and qRT-PCR analysis of MDM2, MDMX, epithelial and mesenchymal markers, and cell migration in **A–D.** human breast MCF10A cells stably transfected with empty vector (EV) or oncogenic K-RasV12; **E–H.** murine prostate cancer cell lines; and **I.** BPH-1 cells. (A, E, I) In representative blots, the full-length MDM2 protein product is marked by an arrow, a faster-migrating product observed in human cells is marked by an asterisk; relative protein expression was evaluated by measuring OD. (B-C, F-G) PCR data represent mean ± SEM. (B–C) *P < 0.05; **P < 1.10^−3^ to MCF10A EV, *n* = 4; (F–G) **P < 1.10^−3^ to cE2 cells, *n* = 4. (D, H) Migration results through an uncoated 8 μm-pore transwell represent the average number (mean ± SD) of migrating cells in five independent viewing fields after 6 h of migration, from 3 independent experiments in technical duplicate; *P < 0.01. Photographs show results of a representative experiment at 40× magnification. (I) Both TGF-β1-induced EMT and MDM2 downregulation are reversible. Cells were treated with vehicle or TGF-β1 for 96 h, passaged and cultivated in the presence or absence of TGF-β1 for another 96 h. The graph shows the fold-over control of normalized protein expression quantified by measuring the OD, *P < 1.10^−2^ to untreated cells, *n* = 3.

Cancer transformation by the Ras oncogene is accompanied by EMT promoting effects [25, 26]. An EMT-associated switch in MDM2 and MDMX expression was observed in benign and K-Ras-transformed MCF10A human breast cells (Figure 2A–2D, Supplementary Figure S2A) [27]. Mouse CaP cell lines with biallelic *PTEN* deletion represent another model of epithelial and mesenchymal cells with similar genetic background (Figure 2E–2H) [28]. Cell lines E2 and E4 expressing mesenchymal markers were derived from androgen-dependent primary tumors in mice, while cell lines cE1 and cE2, manifesting epithelial characteristics, were isolated from recurrent tumors after castration. The epithelial phenotype was again accompanied by increased expression of MDM2.

The TGF-β cytokine is one of the most potent inducers of EMT in cell culture. Consistent with our findings in benign and transformed prostate cell lines (BPH-1, CAFTD01, CAFTD03, and RWPE-1) as well as in breast cell lines (MCF10A), treatment of cells with TGF-β1 for 96 h induced downregulation of E-cadherin, upregulation of mesenchymal markers, and downregulation of MDM2 (Supplementary Figure S2B–S2C). Concomitant upregulation of MDMX was not observed, suggesting that MDM2 and MDMX are regulated independently under EMT-inducing conditions.

EMT is a reversible process; therefore, we tested the reversibility of MDM2 regulation by TGF-β1. BPH-1 cells were cultured in the presence or absence of TGF-β1 for 96 h, which led to the upregulation of mesenchymal markers and downregulation of MDM2 and E-cadherin. The cells were subsequently re-seeded and cultured in the absence of TGF-β1 for another 96 h, which led to reversion of changes in the expression of EMT markers and MDM2 (Figure 2I). On the other hand, prolonged cultivation with TGF-β1 for another 4 d further downregulated both E-cadherin and MDM2 expression. These data show that both TGF-β1-induced downregulation of MDM2 and TGF-β1-induced EMT are reversible phenomena and suggest a mutual association.

### EMT/MET phenotype is associated with changes in MDM2 and MDMX expression in clinical prostate and breast cancer samples

To evaluate MDM2 and MDMX expression with EMT in clinical cancer samples, we analyzed archived formalin-fixed paraffin-embedded tissue specimens from a cohort of prostate cancer (CaP) and breast cancer (BrCa) patients who underwent surgical resection of the primary tumor and its lymph node (LN) metastases. The specimens therefore represent clinical material from different stages of disease in individual patients, allowing relevant evaluation of cancer progression and cellular plasticity.

**Figure 3:**
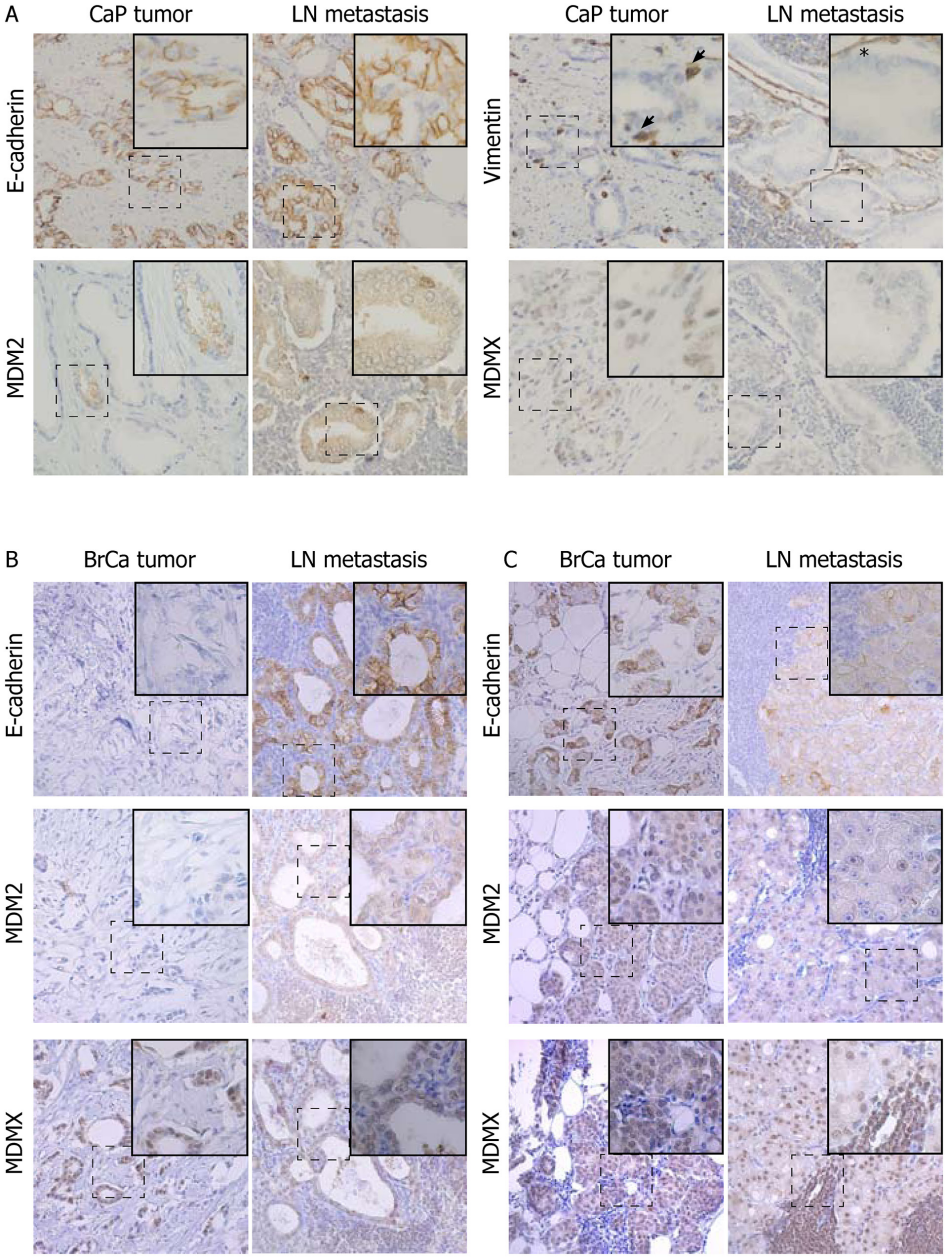
Changes in MDM2 and MDMX protein expression correlate with EMT in a proportion of paired patient prostate and breast tumors and metastases Paired patient samples from primary tumors and LN metastases were stained with the indicated antibodies. LN metastases of CaP **A.** exhibit a more epithelial phenotype compared to the primary tumor from the same patient. Arrows show cytosolic vimentin staining indicating EMT; asterisk shows vimentin positivity in the stroma. LN metastases of BrCa demonstrate both MET-like **B.** and EMT-like **C.** transitions. Magnification: 20×; dashed boxes indicate region in insets at 40× magnification.

In CaP patients, 13 of 16 LN metastases were characterized by MET-like transition, demonstrated by increased expression of epithelial markers (E-cadherin) and lower expression of mesenchymal markers (Slug and vimentin localized in the cytosol of cells with epithelial morphology) (Supplementary Figure S3A). Based on the same markers, most tumor disseminations into seminal vesicles (SV) did not exhibit clear EMT or MET pattern. In LN metastases, MDM2 expression was slightly increased, while MDMX was significantly decreased (Figure 3A, Supplementary Figure S3A). Analysis of paired tumor - metastasis samples from individual patients indicated that MDM2 overexpression or MDMX underexpression was associated with the epithelial phenotype in 50 and 75% of CaP - LN pairs, respectively (Table 1). p53 manifested a positive correlation with MDM2 and MDMX expression (Supplementary Table S1). However, high positivity of p53 expression (>20% of cell nuclei) in 34% of prostate samples indicated defects in p53 function, suggesting that in a proportion of patients, MDM2 and MDMX expression can be regulated independently of p53.

**Table 1:** Changes in MDM2 and MDMX expression clustered by changes in E-cadherin expression in paired CaP and BrCa samples and respective LN metastases

CaP – LN pairs	↓ E-cadherin in metastasis # of pairs	↑ E-cadherin in metastasis # of pairs	Total # of pairs
Total	3	13	16
↑ MDM2 in metastasis	1	7	8
↓ MDM2 in metastasis	1	4	5
→ MDM2 in metastasis	1	2	3
↑ MDMX in metastasis	0	0	0
↓ MDMX in metastasis	3	12 *	15
→ MDMX in metastasis	0	1	1
**BrCa - LN pairs**			
Total	9	9	18
↑ MDM2 in metastasis	3	3	6
↓ MDM2 in metastasis	3	2	5
→ MDM2 in metastasis	3	4	7
↑ MDMX in metastasis	6	1	7
↓ MDMX in metastasis	3	5	8
→ MDMX in metastasis	0	3	3

The table summarizes numbers of cases in specified categories; *, *P* < 0.0005 by Cochran's Q test. Cases in grey boxes correlate with *in vitro* observations

In 18 BrCa patients, both EMT- and MET-like changes were evenly represented in the cohort (Figure 3B–3C). High p53 positivity was observed in 37% of samples. Increased MDMX expression was again associated with the mesenchymal phenotype in 61% of pairs, while decreased MDM2 expression correlated with EMT-like changes in 33% of pairs (Table 1). Moreover, a positive correlation between MDMX and vimentin expression was found in both prostate and breast cohorts, further supporting an association of elevated MDMX expression with the mesenchymal phenotype (Supplementary Table S1).

Altogether, data obtained from clinical specimens show that in CaP and BrCa patients, EMT and MET-like changes accompany the formation of LN metastasis and that overexpression of MDM2 and underexpression of MDMX together with the prevalence of epithelial phenotype in metastatic lesions is the most frequently observed phenomenon.

### Modulation of MDM2 expression affects migration of BPH-1 and CAFTD03 cells

**Figure 4:**
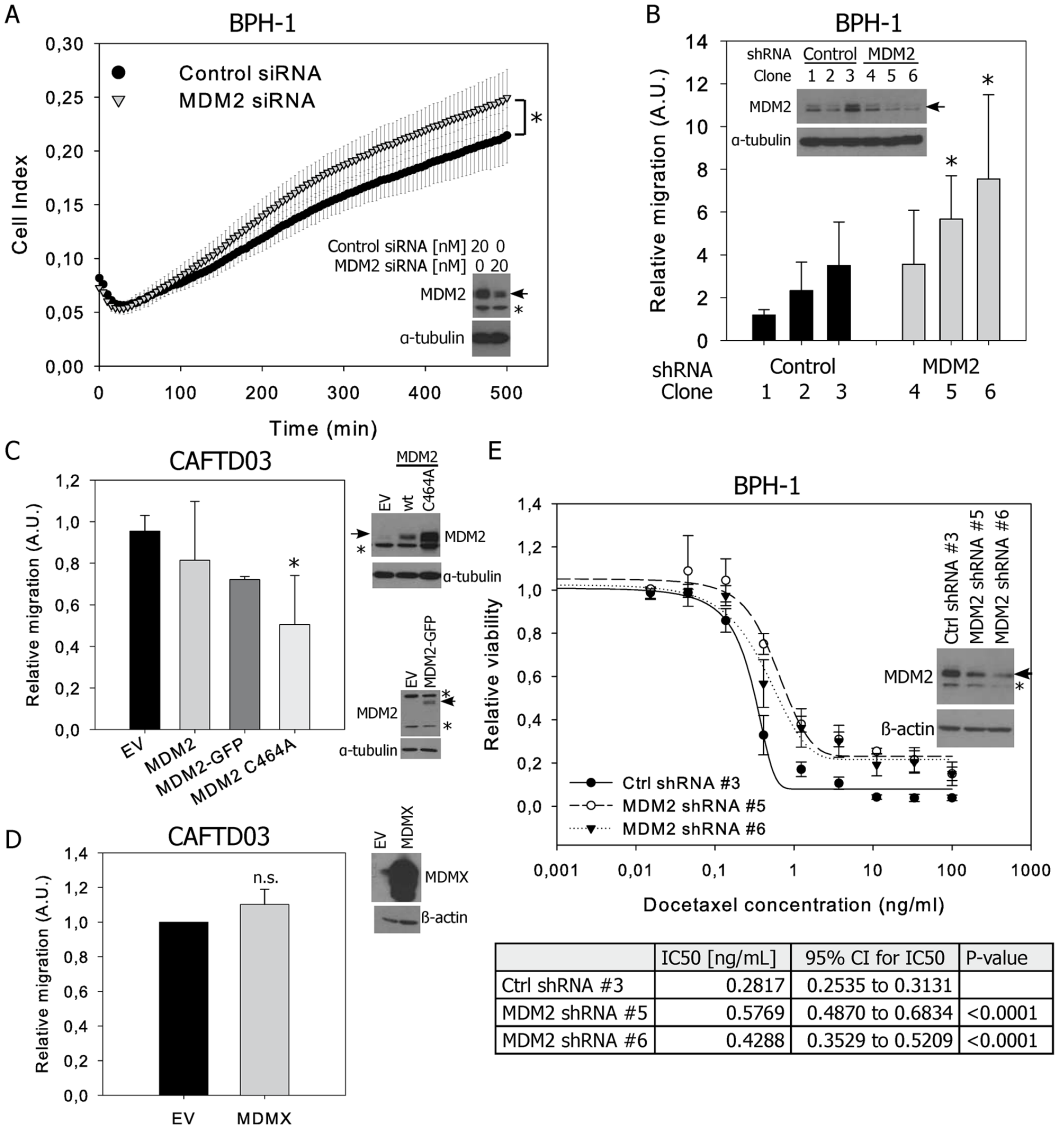
MDM2 expression affects migration and docetaxel sensitivity of prostate epithelial cells **A.** BPH-1 cells were transiently transfected with control or MDM2-specific siRNA and cell migration was evaluated by monitoring the impedance signal using a fibronectin-coated CIM module on an xCELLigence DP system. The graph shows the average Cell Index values (mean ± SD) from three independent experiments, *P < 0.05. **B.** Migration results through an uncoated 8 μm-pore transwell from three independent BPH-1 clones stably transfected with control or MDM2-specific shRNA. **C, D.** Transwell migration of a pool of CAFTD03 cells transiently overexpressing (C) wt, GFP-tagged or the C464A mutant of MDM2 or (D) MDMX. Graphs represent the relative numbers of migrating cells (mean ± SD) from 3 independent experiments in technical duplicate; *P < 0.05; n.s., *P* > 0.05. EV, empty vector. **E.** Viability of stable shRNA-expressing clones from BPH-1 cells treated with docetaxel for 72 h was analyzed using a luminescence-based ATP assay. Graphs show the mean ± SD from 3 independent experiments; IC50 values were calculated from 3 independent experiments.

As decreased MDM2 expression in CAFTD cells compared to the parental BPH-1 cell line correlates with a shift of cell phenotype towards EMT, we analyzed the effect of downregulation or overexpression of MDM2 on chemotactic migration of cells through 8 μm-pore membranes. Even low migration potential of epithelial BPH-1 cells could be measured using label free kinetic analysis of transmembrane migration on an xCELLigence system. Transient or stable MDM2 silencing in BPH-1 cells slightly enhanced cell migration potential (Figure 4A–4B), while overexpression of MDM2 in the CAFTD03 tumorigenic clone led to a moderate inhibition of cell migration (Figure 4C). Interestingly, expression of the ubiquitin ligase-defective MDM2 C464A mutant, which is expressed at higher levels than wt or GFP-tagged MDM2 due to a lack of self-ubiquitination, inhibited CAFTD03 migration more efficiently than wt MDM2 (Figure 4C). On the contrary, MDM2 overexpression induced migration in DU-145 cells bearing a p53 mutation; this effect was partially dependent on MDM2 E3 ligase activity (Supplementary Figure S4A) and suggests that effects of MDM2 modulation on cellular motility are cell type and context specific. No effect of MDMX overexpression on cell migration was observed in CAFTD03 (Figure 4D) or BPH-1 cells (Supplementary Figure S4B). Modulation of MDM2 or MDMX expression did not significantly affect cell proliferation (Supplementary Figure S4C–S4D) and expression of epithelial and mesenchymal markers or EMT regulators at the protein level (data not shown). However, we confirmed an association between lower expression of MDM2, EMT and lower sensitivity to docetaxel observed in Figure 1E in a functional experiment, showing that stable knockdown of MDM2 in BPH-1 cells desensitized them to docetaxel treatment (Figure 4E). These data demonstrate that expression of MDM2 influences important characteristics of cancer cells undergoing EMT.

### MDM2 expression in BPH-1 and CAFTD clones is regulated dominantly from the MDM2 P2 promoter

As modulation of MDM2 and not MDMX expression affected cell migration, we studied potential mechanisms of MDM2 regulation in BPH-1 and CAFTD clones. Decreased *MDM2* mRNA expression in cells expressing mesenchymal markers or after TGF-β1 treatment suggests that transcriptional control is involved in the downregulation of MDM2 accompanying EMT (Figure 1D, Supplementary Figure S2C).

**Figure 5:**
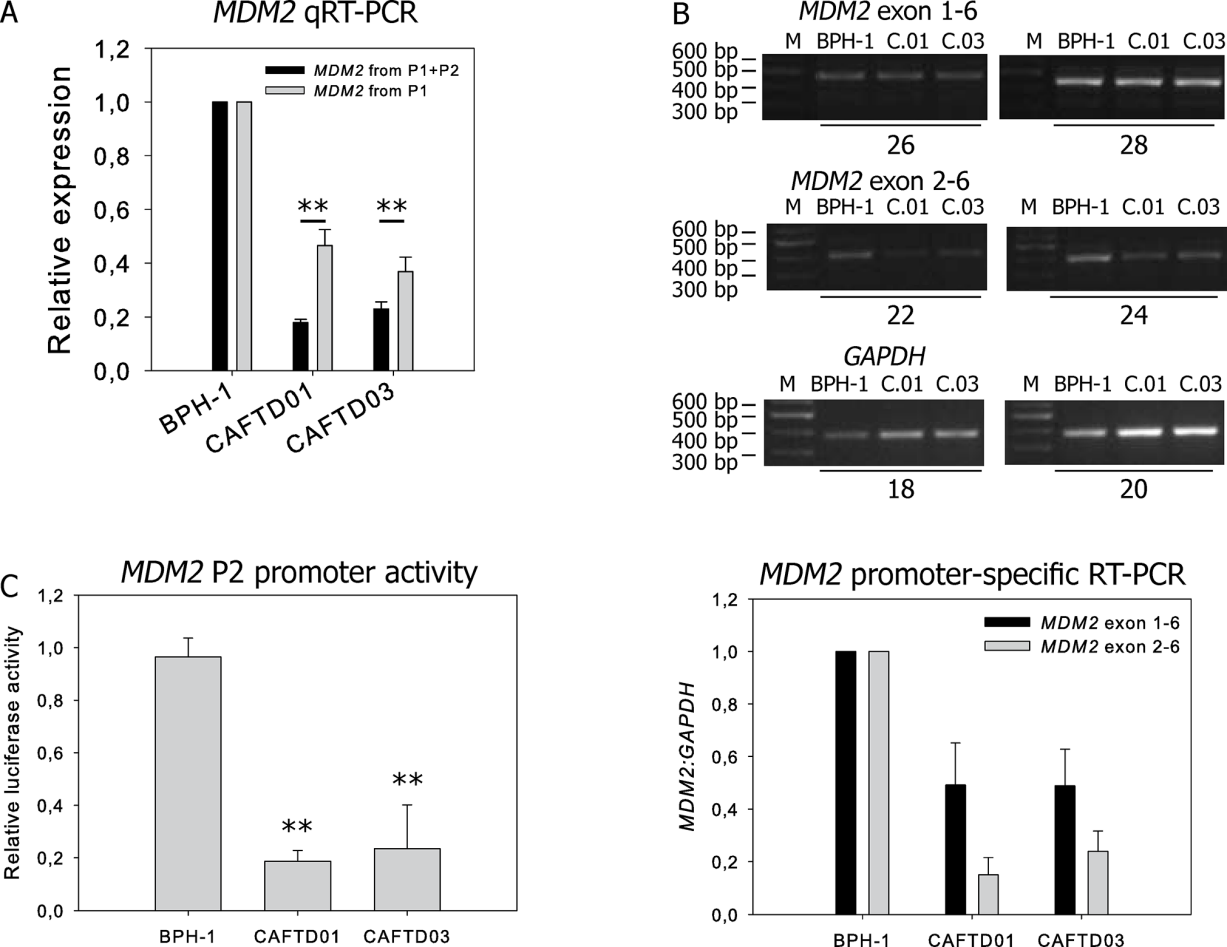
Downregulation of MDM2 in CAFTD clones is driven by transcriptional regulation of two MDM2 promoters **A.** qRT-PCR analysis of MDM2 transcripts from untreated BPH-1 cells and two independent CAFTD clones. The assay design represented by black bars detects mRNA products originating from both P1 and P2 promoters, while the assay design represented by grey bars detects only the product of the P1 promoter. **P < 1.10^−3^ to BPH-1 cells, *n* = 4 **B.** Expression of MDM2 transcripts from the P1 and P2 promoter in BPH-1 cells and tumorigenic CAFTD clones was analyzed by semi-quantitative RT-PCR. Sizes of the PCR products are 452 bp for Exon 1-Exon 6 of MDM2, 398 bp for Exon2-Exon 6 of MDM2, and 372 bp for GAPDH, an internal normalization control. The number of amplification cycles is indicated at the bottom of the gel. The graph represents average relative amounts of respective MDM2 transcripts normalized to GAPDH from four independent experiments. C.01 and C.03 refer to the CAFTD clones. **C.** The activity of the MDM2 P2 promoter was evaluated using a luciferase reporter assay. The cells were transfected with a construct bearing the MDM2 P2 promoter sequence upstream of the firefly luciferase gene, along with a *Renilla* luciferase internal control (pCIneo RL). The graph represents the mean ± SD of normalized luciferase activity, **P < 1.10^−4^ to BPH-1 cells, *n* = 5.

Transcripts of human *MDM2* can be initiated either at the constitutive P1 promoter, or at the p53-responsive P2 promoter, both of which are eventually translated to generate a full-length MDM2 protein. In P1 transcripts, exon 2 is removed by splicing, while P2 transcripts contain exon 2, but lack exon 1. Quantitative reverse transcription polymerase chain reaction (RT-PCR) with primers that detect both transcripts (exon boundary 6–7) showed a more pronounced decrease of *MDM2* mRNA in CAFTD clones, compared to parental cells, than primers designed to detect only the P1 product (exon boundary 1–3) (Figure 5A). Semi-quantitative RT-PCR analysis using primers that discriminate between the P1 and P2 products also suggests a more robust difference in transcription from P2 between BPH-1 cells and CAFTD clones (Figure 5B). Moreover, the luciferase activity of a reporter construct encompassing the P2 promoter was significantly decreased in both CAFTD clones (Figure 5C). Analysis of mRNA stability after inhibition of translation by actinomycin D did not reveal any differences in the stability of *MDM2* or *MDMX* transcripts between the clones (Supplementary Figure S5A).

Decreased MDM2 expression in CAFTD clones cannot be attributed to decreased protein stability, because the half-life of MDM2 in CAFTD01 and CAFTD03 clones (75 and 90 min, respectively) was even higher than that in BPH-1 cells (35 min) (Supplementary Figure S5B). Inhibition of proteasomal degradation by MG-132 did not reveal any significant differences in the MDM2 degradation rates between the cell lines (Supplementary Figure S5C). These results suggest that decreased MDM2 expression in the CAFTD clones results from altered transcriptional regulation, mainly from the P2 promoter.

The *MDM2* P2 promoter contains p53 binding sites, which prompted us to address the question of p53 expression and function in our *in vitro* models. Two p53 target genes were selected for expression analysis, p21^Cip1/Waf1^ which is implicated in the inhibition of cell cycle progression [29], and a TGF-β family cytokine, GDF-15, which exerts multiple roles in the stress response and was proposed as a marker of p53 pathway activation [30] [31]. MDM2 levels correlated with expression of p53 and its downstream targets in p53 wt MCF10A cells and in murine cell lines E2, E4, cE1 and cE2 (Supplementary Figure S6B–S6C). However, in SV40-transformed cells BPH-1 and CAFTD, trends in MDM2 expression did not correspond with the expression of p53, p21 or GDF-15 (Supplementary Figure S6A).

p53 mutations were not detected in BPH-1 or CAFTD03 cells using a functional screening approach (data not shown) [32]. However, due to SV40 T-large antigen immortalization, the ability of p53 to induce its transcriptional target, *CDKN1A*, encoding the p21^Cip1/Waf1^ protein upon DNA damage by gemcitabine, was attenuated in BPH-1 and CAFTD03 cells, compared to the effects on wt p53-containing MCF10A cells (Supplementary Figure S6D–S6E). On the other hand, co-immunoprecipitation analysis indicated that the physical interaction between p53 and MDM2 was preserved in CAFTD03 cells (Supplementary Figure S6F). The fact that MDM2 modulation accompanying EMT is observed in cellular models with both normal and immortalization-affected p53 suggests that the regulation of MDM2 basal levels does not require intact p53 function.

### Intact p53 function is dispensable for regulation of MDM2 expression by Slug and Twist in CAFTD03 cells

**Figure 6:**
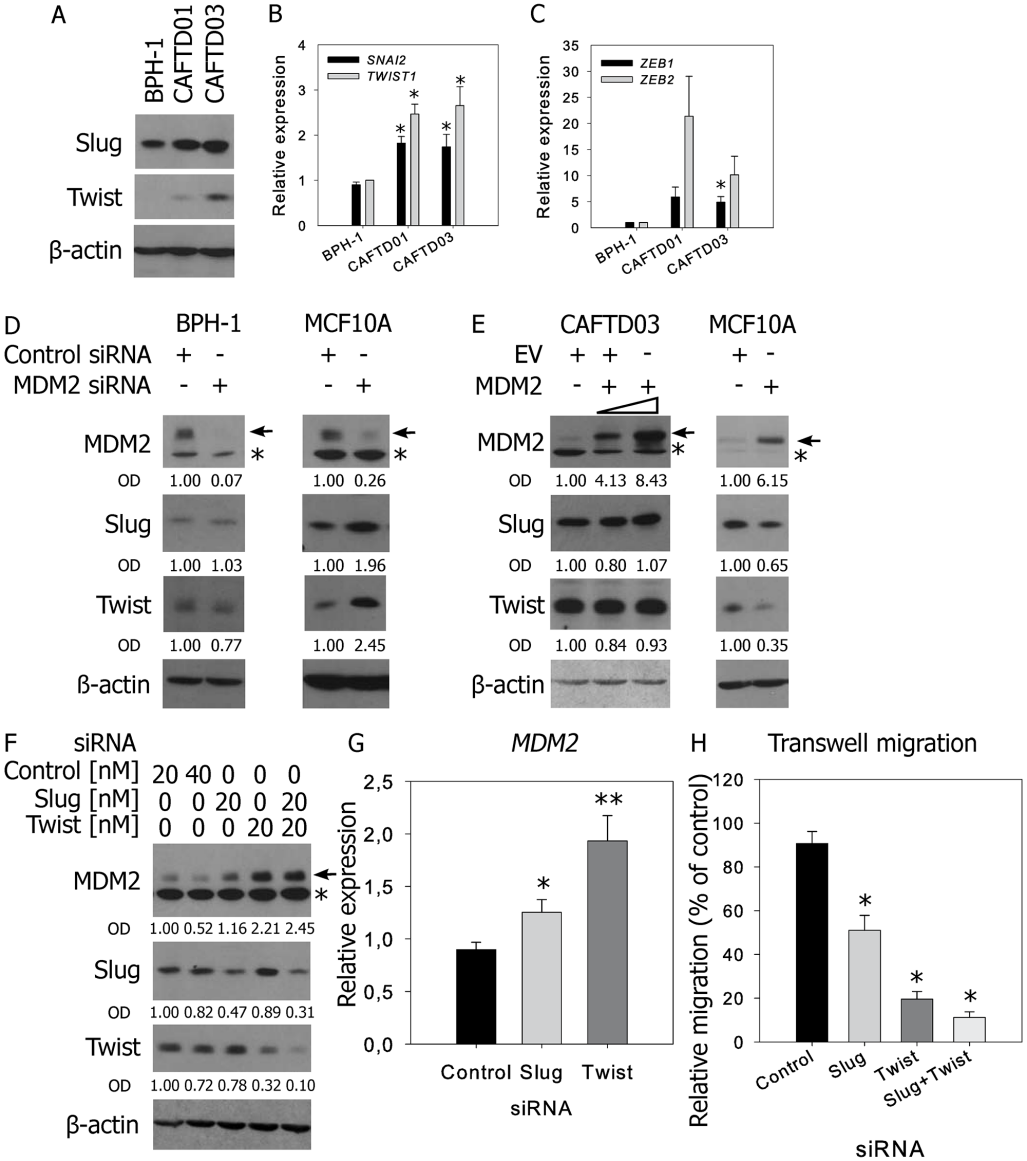
Regulation of Slug by MDM2 requires intact p53 function, while downregulation of Slug or Twist in CAFTD03 cells upregulates MDM2 and inhibits cell migration **A–C.** Western blotting (A) and qRT-PCR (B–C) analysis of EMT-driving transcription factors in BPH-1 and CAFTD cells. PCR data represent mean ± SEM of at least three independent experiments, *P < 0.05 to BPH-1 cells. **D–E.** BPH-1 and MCF10A cells were transfected with (D) MDM2-specific siRNA or (E) increasing concentrations of MDM2 overexpressing plasmid and expression of MDM2, Slug and Twist was evaluated by western blotting 48 h after transfection. **F–H.** CAFTD03 cells were transfected with siRNA against Slug, Twist, or both. Protein expression was evaluated by (F) western blotting and (G) mRNA expression by qRT-PCR 48 h after transfection. The full-length MDM2 protein product is marked by an arrow; a faster-migrating product is marked by an asterisk. (H) Transwell migration of CAFTD03 cells transfected with 20 nM siRNA. Bars show the mean ± SD values for relative cell migration of three independent experiments, *P < 0.05; **P < 0.01.

MDM2 was reported to cooperate with p53 as a negative regulator of the EMT-inducing transcription factor Slug [11]. In both sets of prostate cell lines, expression of Slug was increased in cells expressing mesenchymal markers (Figure 6A–6B, Supplementary Figure S7A). In all sets of cell lines, expression of mesenchymal markers correlated with increased expression of the transcription factors Twist1 or Twist2, ZEB1, and ZEB2, which suggests that multiple transcription factors may participate in the induction and maintenance of the EMT phenotype in these cellular cancer models (Figure 6A–6C, Supplementary Figure S7A–S7B).

Analysis of Slug protein stability by cycloheximide treatment showed a slower degradation rate of Slug in CAFTD clones, compared to that in BPH-1 cells (Supplementary Figure S7C), while proteasome inhibition led to a more pronounced stabilization of Slug in BPH-1 cells (Supplementary Figure S7D). These data suggest that Slug expression is controlled at the level of both transcription (Figure 6B) and protein stability (Supplementary Figure S7D), implicating a putative role of the ubiquitin-proteasome pathway in the regulation of Slug. However, downregulation of MDM2 in BPH-1 cells or overexpression of MDM2 in CAFTD03 cells did not induce any changes in expression of Slug, Twist (Figure 6D–6E), or epithelial/mesenchymal markers (data not shown). On the other hand, MDM2 silencing in MCF10A cells upregulated both Slug and Twist, and MDM2 overexpression downregulated Slug and Twist in these cells (Figure 6D–6E). These data confirm that proper p53 function is crucial for MDM2-mediated ubiquitination of Slug and show that Slug stability is not affected by MDM2 in immortalized cells with wt p53 expression, but compromised p53 transcriptional response (Supplementary Figure S6D–S6E).

Based on abundant and differential expression of Slug and Twist in BPH-1 cells and tumorigenic CAFTD clones (Figure 6A–6B), we hypothesized that these EMT-inducing transcription factors can regulate MDM2 expression. Individual or combined silencing of Slug and Twist using siRNA-mediated methods induced MDM2 expression in CAFTD03 cells. This effect was observed both at the protein and mRNA level, confirming a role of transcription repressors in the regulation of MDM2 (Figure 6F–6G, Supplementary Figure S7E). Importantly, individual and combined silencing of Slug and Twist significantly inhibited cell migration (Figure 6H, Supplementary Figure S7F). Therefore, Slug and Twist are implicated not only in the regulation of cell motility, but can also function as regulators of MDM2 expression, thereby linking the regulation of MDM2 expression to EMT in tumorigenic prostate epithelial cells.

## DISCUSSION

In this study, we showed that in the context of certain CaP and BrCa subtypes and in multiple *in vitro* models, downregulation of MDM2 and upregulation of MDMX accompany EMT-like changes and that modulation of MDM2 affects cell migration. The association of MDM2 with cellular motility and the fact that reciprocal changes in MDMX expression were not observed in all *in vitro* models prompted us to concentrate on the mechanisms of MDM2 expression control.

MDM2 expression and stability can be affected by multiple previously published mechanisms [33, 34]. In case of BPH-1 and CAFTD cells, we can rule out an effect of epitope masking by phosphorylation [33], as MDM2 protein can be detected by several phosphorylation-insensitive monoclonal antibodies (Supplementary Figure S1C–S1D). From the comparable stability of MDM2 protein in BPH-1 cells and CAFTD clones and the increased activation of the Akt signaling pathway in CAFTD clones (Supplementary Figure S2D, [24]), we surmised that Akt-phosphorylation-mediated MDM2 stabilization via decreased ubiquitination [34] is unlikely to contribute to the mechanism of MDM2 regulation in our cancer models. On the contrary, we found that MDM2 levels are controlled at the transcriptional level, implicating the role of transcription regulators in MDM2 expression.

As an alternative model of EMT induction, we show that TGF-β-induced downregulation of MDM2 is reversible, its kinetics correlates with E-cadherin downregulation (data not shown), and it is mirrored in the downregulation of the MDM2 transcript. Our model does not recapitulate findings from other reports that correlate MDM2 expression with enhanced activation of TGF-β signaling pathway or propose an MDM2-based mechanism of TGF-β resistance [35, 36]. On the contrary, lower MDM2 expression in CAFTD clones is associated with increased phosphorylation of Smad2 (Supplementary Figure S2D) and resistance to antiproliferative effects of TGF-β [24].

The observation that the increased protein level of MDMX by CAFTD03 compared to BPH-1 is not proportional to the RNA level (Figure 1A, 1D) suggests that elevated MDMX level in CAFTD03 cells can result from a decrease in MDM2 E3 ligase activity towards MDMX [37]. MDMX itself does not have any significant effect on migration of BPH-1 or CAFTD cells, while MDM2 can modulate cell migration independently of its E3 ligase activity. Although MDMX did not influence cell motility in our model, it can exert other pro-tumorigenic activities such as promotion of cell proliferation and tumor growth [38].

Our analysis of paired samples from primary tumor and metastases from the same patient represents a unique opportunity for delineating the EMT pattern during cancer progression, due to the fact that analysis of EMT-associated changes in pooled patient cohorts may not necessarily yield reliable data (the effects can be partial, transient, or occur at specific tumor locations such as the invasive front) [2]. In order to obtain sufficient information, the EMT pattern was evaluated based on multiple markers; besides E-cadherin we also analyzed membrane-bound β-catenin as an epithelial marker and vimentin and Slug as mesenchymal markers. Changes in these auxiliary parameters mostly correlated with the trends in E-cadherin expression.

Our finding, MDM2 downregulation in EMT, is corroborated by the fact that MDM2 expression negatively correlates with Twist in liposarcoma [39] and with Slug in a proportion of lung tumors [11]. In a study by Yang *et al.,* a pattern of MDM2 expression intensity corresponding to E-cadherin expression was observed in 46% of breast cancer patients [22]. Furthermore, in colorectal cancer, the presence of liver metastasis was significantly associated with low MDM2 mRNA expression in the primary tumor [18], and MDM2 amplification negatively correlated with occurrence of distant metastases [19]. On the other hand, an extensive study in CaP patients observed a significant association between combined MDM2 and Ki-67 expression and distant metastases [20]. In spite of shorter disease-free survival of invasive ductal breast carcinoma patients with MDM2-expressing tumors, these tumors were significantly smaller and fewer patients with the MDM2-expressing tumors presented with LN metastases [21]. These findings suggest that the effects of MDM2 on invasiveness or metastasis can be tissue- and/or site-specific and the use of MDM2 as a prognostic marker should be combined with other parameters.

Our observation of MDM2 downregulation in EMT differs from that described in other studies, which showed that MDM2 promotes EMT and cell motility [22, 35, 40]. Nevertheless, our data confirm the migration-promoting effects of MDM2 in DU145 cells, which is consistent with the EMT-associated induction of MDM2 in DU145 cells [41]. These data suggest that the role of MDM2 in BPH-1 and CAFTD03 cells could be strictly cell type- and context-specific. Importantly, we confirm the association of EMT phenotype and lower MDM2 expression with a clinically important decrease in sensitivity to docetaxel (Figure 1E, 4E) [6].

We show that higher basal expression of MDM2 in BPH-1 cells can result from the activity of the P2 promoter, which contains elements mediating both p53-dependent and p53-independent effects [42]. Even though Slug or Twist binding sites could not be identified in the MDM2 promoter using bioinformatics tools, Twist may act as a part of hetero-complexes with other transcription factors of the basic helix-loop-helix family interacting with the MDM2 locus [43, 44]. Increased MDM2 expression could also result from higher copy numbers of chromosome 12 fragments fused to other chromosomal fragments in BPH-1 cells, compared to the copy numbers in CAFTD clones [45].

Among multiple EMT-inducing transcription factors, we concentrated on Slug and Twist due to their abundant expression and rapid induction under EMT-inducing conditions in prostate epithelial cells [4]. Although the expression levels of MDM2 in BPH-1 cells and CAFTD clones correlate with the stabilization of Slug, which we described as an early factor responsible for TGF-β-induced EMT in this model [4], we did not find evidence supporting a physical interaction between Slug and MDM2 (data not shown) or direct regulation of Slug by MDM2 E3 ligase activity. Defects in p53 function, induced by p53 protein stabilization in a transcriptionally inactive form may be responsible for a lack of MDM2-mediated regulation of Slug in our *in vitro* model, which is in accordance with a previously reported mechanism of Slug regulation by a fully-active p53-MDM2 complex [11].

**Figure 7:**
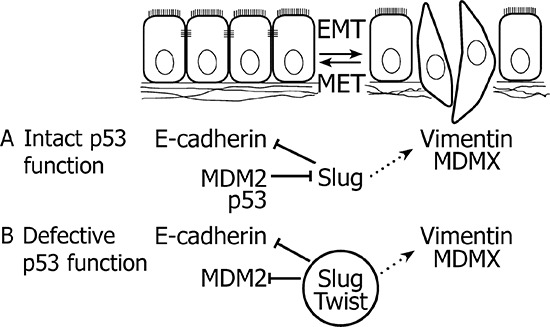
Crosstalk between MDM2, Slug and Twist in EMT **A.** In case of intact p53 function, MDM2 cooperates with p53 in the degradation of Slug, thereby preserving the epithelial phenotype. **B.** When p53 function is impaired, elevated expression of MDM2 correlates with the epithelial phenotype. Slug and Twist inhibit expression of MDM2 and promote EMT, which is associated with upregulated expression of MDMX. Solid lines delineate direct effects, dotted lines represent indirect mechanisms.

Consistent with previous studies, our data show that downregulation of Slug or Twist can suppress cell motility and invasiveness [46]. Notably, we show that these effects of Slug and Twist are accompanied by induction of MDM2 in tumorigenic prostate epithelial cells, which provides a potential link between the regulation of MDM2 and EMT-inducing transcription factors (Figure 7). This phenomenon was observed in the context of defective p53 function, characterized by stabilized p53 that maintains the capacity to interact with MDM2 and MDMX, but its ability to transactivate target genes upon DNA damage is limited. In this specific context, stabilized MDM2 can suppress cell migration and, importantly, may have clinical impact by affecting cell susceptibility to the cytostatic effects of docetaxel, a chemotherapeutic agent routinely used in the treatment of advanced CaP. Our findings may therefore help to elucidate several unexpected observations in clinical cancer samples and propose an alternative context-specific role of MDM2 in EMT, tumor progression, and sensitivity to chemotherapy in cells with defective p53 function.

## MATERIALS AND METHODS

### Cell culture, treatments and transfections

BPH-1 cells [23] and BPH-1-derived tumorigenic clones CAFTD01 and CAFTD03, isolated from tumors obtained from coinjection of BPH-1 cells and human prostatic carcinoma-associated fibroblasts into nude mice [47], were obtained from S.W.Hayward, Vanderbildt University, TN, USA. MCF10A LXSN and LXSN V12 cells [27] were a kind gift from B.H. Park, Johns Hopkins University, MD, USA. All cells were maintained under standard conditions as described previously [4, 48]. The AmpFLSTR® Identifiler® PCR Amplification Kit (Life Technologies) was used to verify the origin of cell lines. TGF-β1 (Millipore, Billerica, MA) was dissolved in PBS containing 2 mg/ml BSA. Docetaxel (Cell Signaling) was dissolved in DMSO and further diluted in PBS. Gemcitabine was dissolved in water and cells were treated with 16.7 nM drug for 24 h. For analysis of mRNA and protein stability, cells were treated with 0.3125 μM actinomycin D, 1 μM cycloheximide or 1 μM MG-132.

A custom synthesized MDM2-specific siRNA (5′ AGG AAU UUA GAC AAC CUG AA dTdT 3′) was obtained from VBC-Biotech. siRNAs targeting Slug (sc-38398) and Twist (sc-38604) were from Santa Cruz Biotechnology. Transient transfection using the X-treme GENE siRNA Transfection Reagent (Roche, 20 nM siRNA) was performed as described previously [4]. BPH-1 cells stably knocked down for MDM2 were generated after transduction with lentiviruses expressing shRNA against MDM2 (sc-29394-V, Santa Cruz Biotechnology), 1 μg/ml puromycin selection, and single-cell cloning.

Plasmids coding for human wt MDM2 (pCHDM1A), MDM2 C464A mutant, Myc-tagged MDMX, and MDM2 P2 promoter luciferase reporter construct (pGL2-MDM2-luc) have been described previously [49–52]. Transfections were performed using the Neon® Transfection System (Life Technologies) according to the manufacturer's recommendations.

### Electrophoresis and western blotting

Preparation of cell lysates and western blotting was performed as described previously [4]. Cell extracts for immunoprecipitation were prepared by scraping cells in monolayer in lysis buffer containing 1% Triton X-100, 25 mM HEPES pH 7.4, 60 mM NaCl, 1 mM EDTA, 1.5 mM MgCl_2_ and protease inhibitors. Immunoprecipitation with antibodies specified in Supplementary Table S2 was performed using standard procedures. Primary and secondary antibodies are specified in Supplementary Table S2. The specificity of MDM2 2A10 antibody was verified by knockdown and overexpression experiments (Supplementary Figure S1B). Analysis of optical density was performed using ImageJ software.

### RNA isolation, RT-PCR, and promoter activity assay

Total RNA was isolated using a High Pure RNA Isolation Kit (Roche) and equal amounts of RNA were reverse transcribed using a High Capacity RNA-to-cDNA Kit (Life Technologies). Quantitative RT-PCR was carried out as described previously [48] using TaqMan assays with primers and conditions specified in Supplementary Table S3. Relative changes in gene expression were calculated using the E-method based on real efficiency values obtained from calibration curves [53, 54]. *MDM2* promoter-specific PCR was performed as described previously [55] and the obtained products were analyzed semi-quantitatively after resolution on a 1% agarose gel. Activity of the *MDM2* P2 promoter was evaluated 48 h after electroporation of cells with a 49:1 mixture of pGL2mdm2-luc [52] and pCI-neo-hRL using the Dual luciferase assay kit (Promega).

### Cell migration assays

The cells were starved overnight in serum-free medium containing 0.1% bovine serum albumin and seeded on uncoated 8 μm-pore Transwell chambers (BD Biosciences) or on CIM plates (Roche) coated with 20 μg/mL fibronectin. Human foreskin fibroblasts (HFF-1 cells; ATCC, SCRC-1041™) conditioned serum-free medium was used as a chemoattractant. Transwell chambers were fixed after 4–6 h in 4% paraformaldehyde and stained with 0.1% crystal violet. Non-migrating cells were removed using a cotton swab and images in five standardized view fields were documented using an Olympus IX70 microscope with an UPLanFl N 4×/0.13 objective. Migrating cells were counted and analyzed using ImageJ software. Alternatively, cell migration was monitored in real-time based on measurement of impedance on an xCELLigence RTCA DP System (Roche). Short intervals of cell migration (less than 6 h) and no significant differences in cell numbers between experimental groups at the moment of transwell seeding rule out off-target effects of proliferation in migration experiments.

### Cell viability assay

Cells plated in 96-well plates were treated with 0.015–100 ng/mL docetaxel and the number of viable cells in culture was quantified by measuring the ATP present after 72 hours using CellTiter-Glo® One Solution Assay (Promega).

### Immunostaining of clinical CaP and BrCa samples

Archival tissue samples of 105 CaPs, 16 lymph node (LN) metastases, 35 tumor outgrowths into seminal vesicles (SV), 33 tumor-adjacent benign prostatic hyperplasias (BPH), and 18 BrCa samples and respective LN metastases were obtained from patients who underwent surgery between the years 1998 and 2011 in the University Hospital Olomouc. The study was approved by the Ethics Committee of the University Hospital and Medical Faculty of Palacky University in Olomouc.

Immunostaining was performed using routine immunostaining procedures with validated antibodies specified in Supplementary Table S2. Specimens were assessed semi-quantitatively using the histoscore (H-score) method (percentage positivity in 10% increments, multiplied by staining intensity [categorized as: 0, absent; 1, weak; 2, moderate; and 3, strong], resulting in a final H-score between 0 [minimum] and 300 [maximum]).

For antibody validation, control cells from *in vitro* culture were homogeneously distributed in an agarose gel matrix, creating an artificial tissue, and then formalin-fixed and paraffin-embedded and stained with MDM2 2A9 antibody using standard immunohistochemical procedures (Supplementary Figure S3B) [56]. Moreover, the MDM2 2A9 and MDMX/MDM4 antibodies were validated previously for staining of human cancer tissues [57–59].

The EMT pattern was compared between primary tumors and their respective LN disseminations based on the difference in the H-score of E-cadherin. Membrane-bound β-catenin, cytosolic vimentin and, in case of CaP, Slug were used as auxiliary parameters, showing appropriate positive or negative correlation with E-cadherin in CaP-LN and BrCa-LN pairs.

### Statistical analysis

The data were analyzed in STATISTICA for Windows (StatSoft) using appropriate statistical tests based on data homogeneity: One-way Analysis of Variance, Student's *t*-test, or Mann-Whitney *U*-test. Data from clinical samples were analyzed by Spearman's correlation, Cochran's Q test and Kruskal-Wallis test. The IC50 values from the viability assays were analyzed using the Extra sum of squares F test.

## SUPPLEMENTARY FIGURES AND TABLES


