# Supplementary material for: Opposite regulation of MDM2 and MDMX expression in acquisition of mesenchymal phenotype in benign and cancer cells
Source: Oncotarget. 2015 Sep 25;6(34):36156–71. doi: 10.18632/oncotarget.5392 (PMC4742168; doi:10.18632/oncotarget.5392)
Supplement: Supplementary file 1 [file oncotarget-06-36156-s001.pdf]

## SUPPLEMENTARY FIGURES AND TABLES

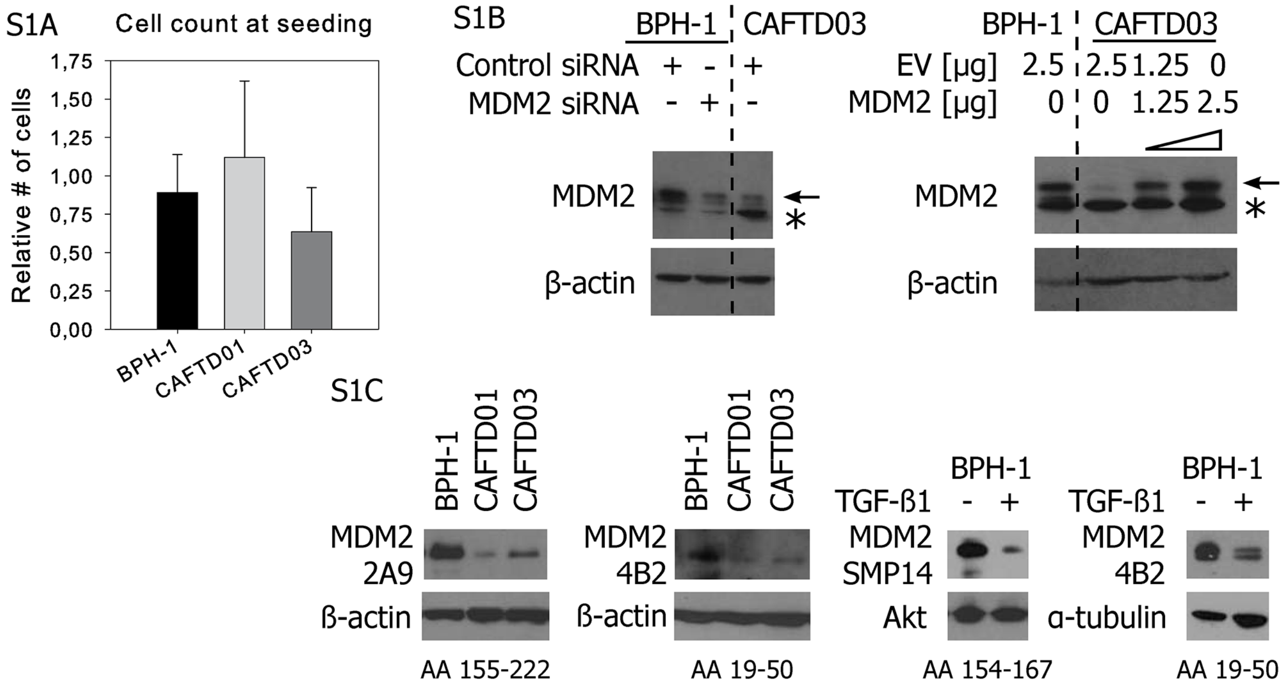


**Supplementary Figure S1: Analysis of cell proliferation in BPH-1 and CAFTD cell lines and validation of MDM2 antibodies. S1A.** The proliferation rate of benign and tumorigenic BPH-1 and CAFTD cells is not significantly different. Cells were cultivated for 48 hours, serum-starved overnight and viable cells were counted prior seeding into Transwell chambers. The graph shows the relative number of cells (mean ± SD) from 4 independent experiments, $P > 0.05$ to BPH-1 cells. **S1B.** Identification of specific and non-specific products of immunoblotting using the MDM2 2A10 antibody. Based on knockdown and overexpression experiments, the specific MDM2 product marked by an arrow is distinguished from a non-specific faster migrating product marked by an asterisk. **S1C.** Downregulation of MDM2 expression does not result from epitope masking by phosphorylation. The effect of MDM2 downregulation in CAFTD cells or after TGF-β1 treatment was observed using both phosphorylation sensitive (SMP-14) and phosphorylation insensitive (2A9, 4B2) monoclonal antibodies. Epitope locations of antibodies are indicated below.

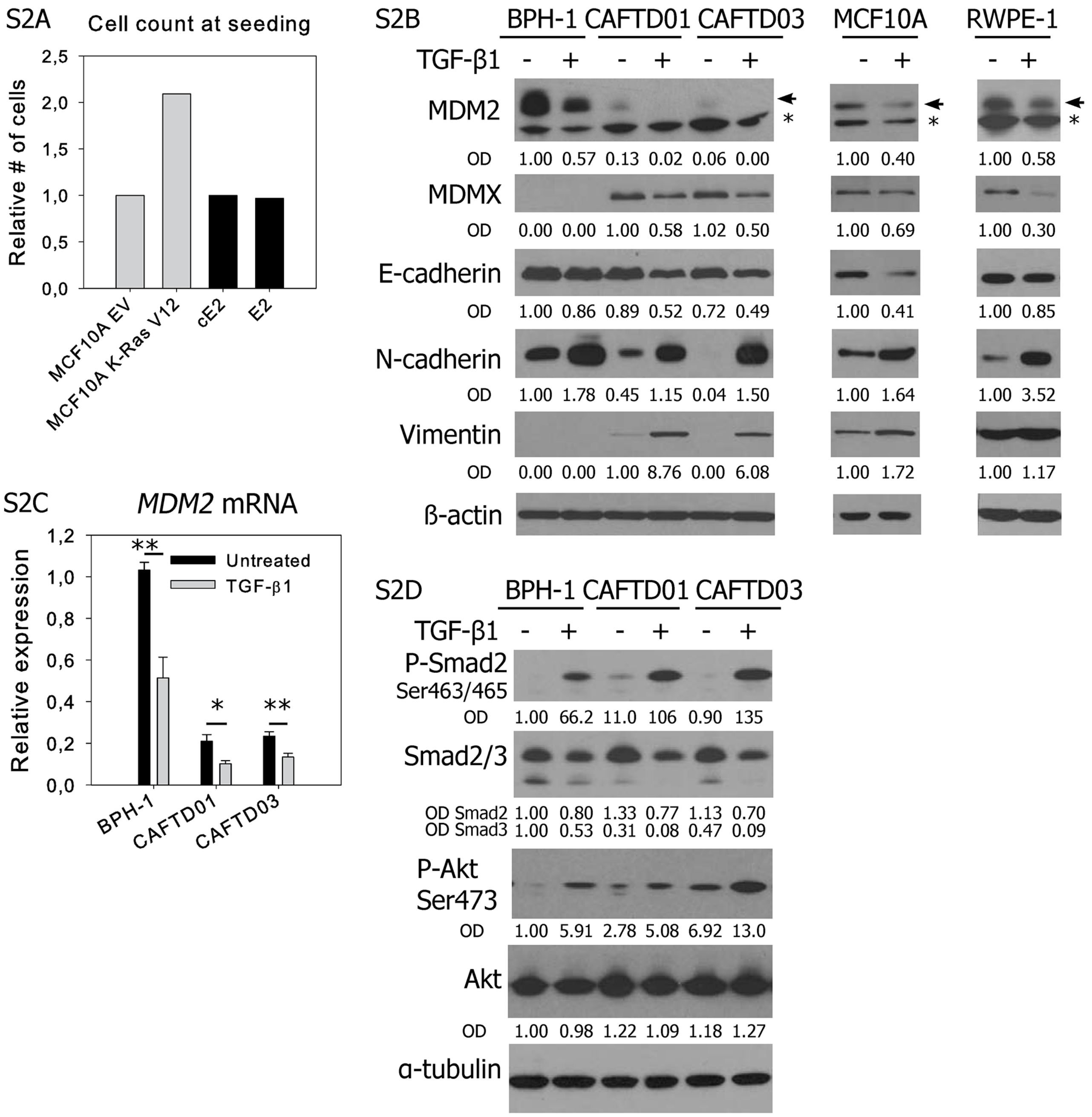


**Supplementary Figure S2: Analysis of cell proliferation in prostate and breast cell lines with distinct EMT status and expression of MDM2, MDMX and EMT markers in a model of TGF-β1-induced EMT. S2A.** The proliferation rate of cells in migration experiments. Cells were cultivated for 48 hours, serum-starved overnight and viable cells were counted prior seeding into Transwell chambers. The graph shows average cell number from 2 independent experiments, $P > 0.05$. **S2B–S2C.** Expression of MDM2, MDMX, and epithelial and mesenchymal markers in the indicated cell lines was evaluated 96 h after treatment of cells with 10 ng/mL TGF-β1. In western blotting (S2B), the full-length MDM2 protein product is marked by an arrow; a faster-migrating product is marked by an asterisk. PCR data (S2C) represent mean ± SEM; $*P < 5.10^{-2}$, $**P < 1.10^{-2}$ to BPH-1 cells, $n = 7$. **S2D.** Phosphorylation of Smad2 and Akt is enhanced in CAFTD cells compared to BPH-1 in both untreated and 10 ng/mL TGF-β-treated cells.

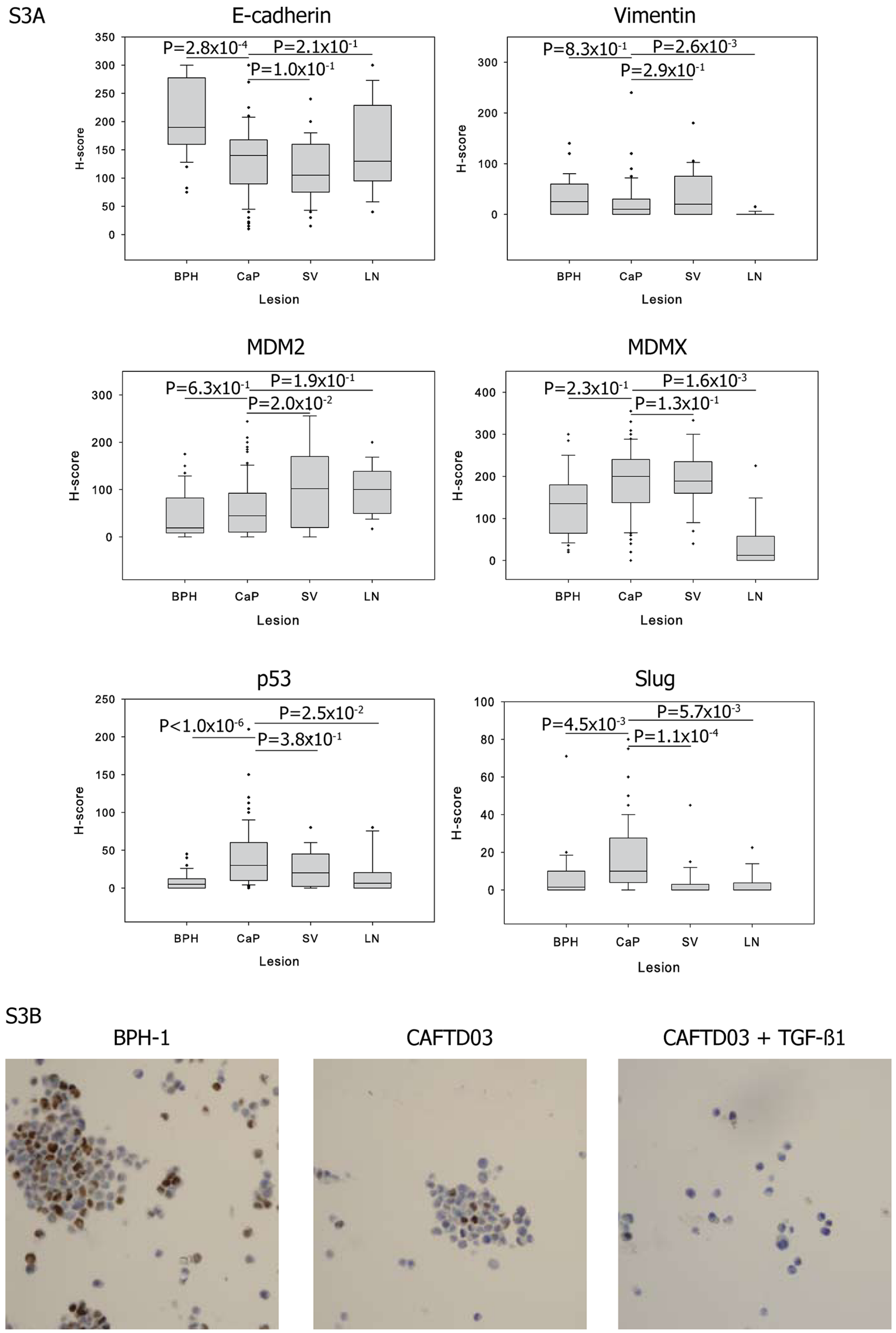


**Supplementary Figure S3: Changes in MDM2 and MDMX protein expression correlate with EMT in a proportion of clinical prostate tumors and metastases. S3A.** Cancer transformation in the prostate is accompanied by EMT, while LN metastases exhibit an MET phenotype in unpaired patient samples. Box plots show H-scores in 4 distinct regions of patient samples: BPH – non-tumor tissue, *n* = 33; CaP – prostate carcinoma, *n* = 101; SV – invasions into seminal vesicles, *n* = 35; LN – lymph node metastases, *n* = 18. Statistical significance of expression differences was evaluated using the Kruskal-Wallis test. **S3B.** Validation of MDM2 2A9 antibody on artificial tissues from prostate cell lines. In accordance with the results of western blotting and qRT-PCR, MDM2 expression in the clone CAFTD03 is lower than in BPH-1 cells and it is further decreased by TGF-β1 treatment. Magnification: 20×.

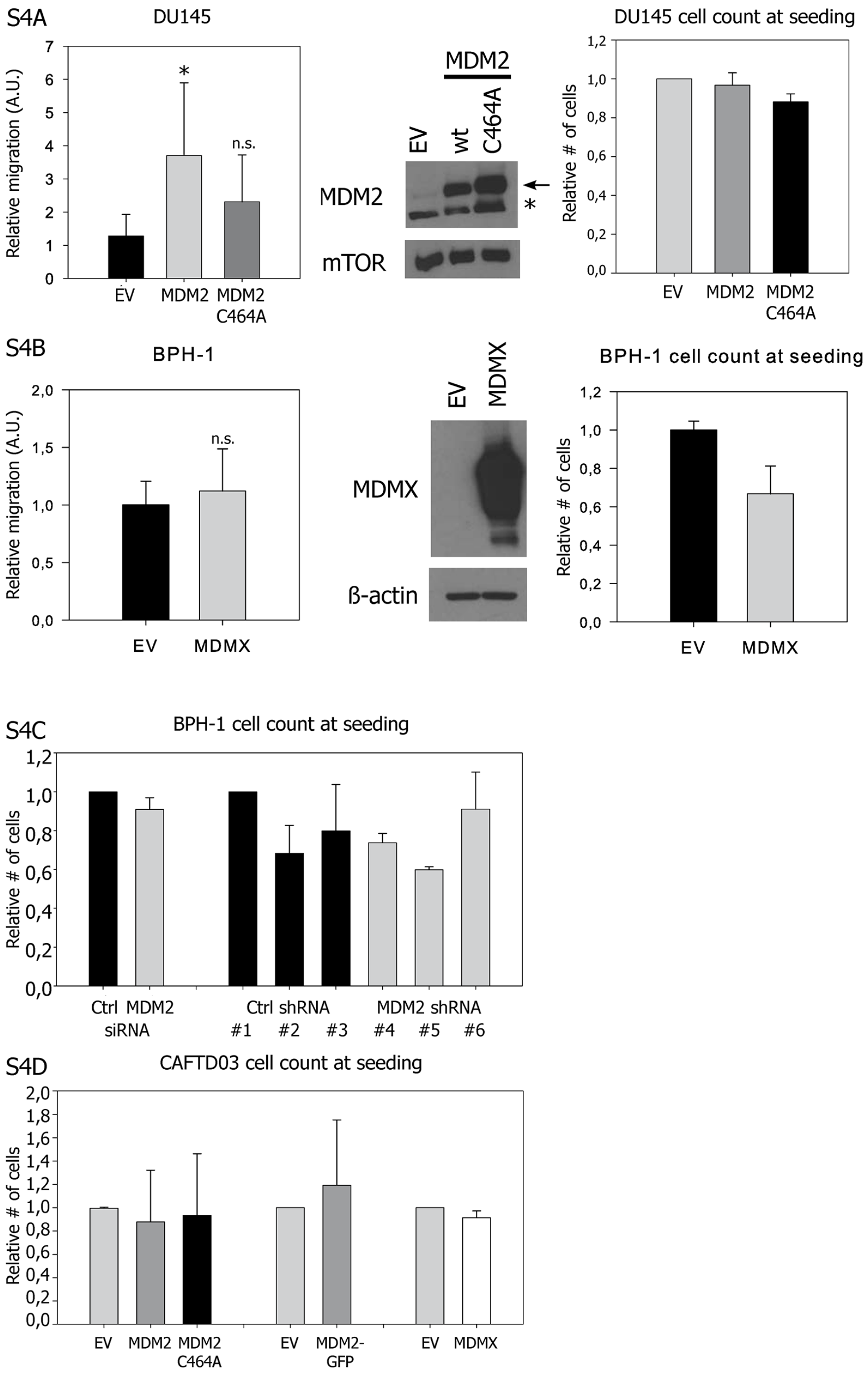


**Supplementary Figure S4: MDM2 expression affects migration of prostate epithelial cells without significant effects on cell proliferation. S4A.** Migration results through an uncoated 8 µm-pore transwell of a pool of DU145 cells transiently overexpressing the indicated MDM2 construct. The graphs represent mean ± SD from three independent experiments in technical duplicate; *$P < 0.05$; n.s., $P > 0.05$. The signal corresponding to the specific product is marked by an arrow; a faster migrating endogenous product is marked by an asterisk. EV, empty vector; mTOR, mammalian target of rapamycin detection serves as a control of equal loading. No significant difference in cell count at transwell seeding indicates that MDM2 does not affect cell proliferation. **S4B.** Migration results through an uncoated 8 µm-pore transwell of a pool of BPH-1 cells transiently overexpressing MDMX. The graphs represent mean ± SD from one experiment in technical duplicate; n.s., $P = 0.57$. **S4C–S4D.** The proliferation rate of cells in migration experiments. Transfected cells were cultivated for 24 hours, serum-starved overnight and viable cells were counted prior seeding into Transwell chambers. The graphs show average cell number from at least 3 independent experiments, $P > 0.05$.

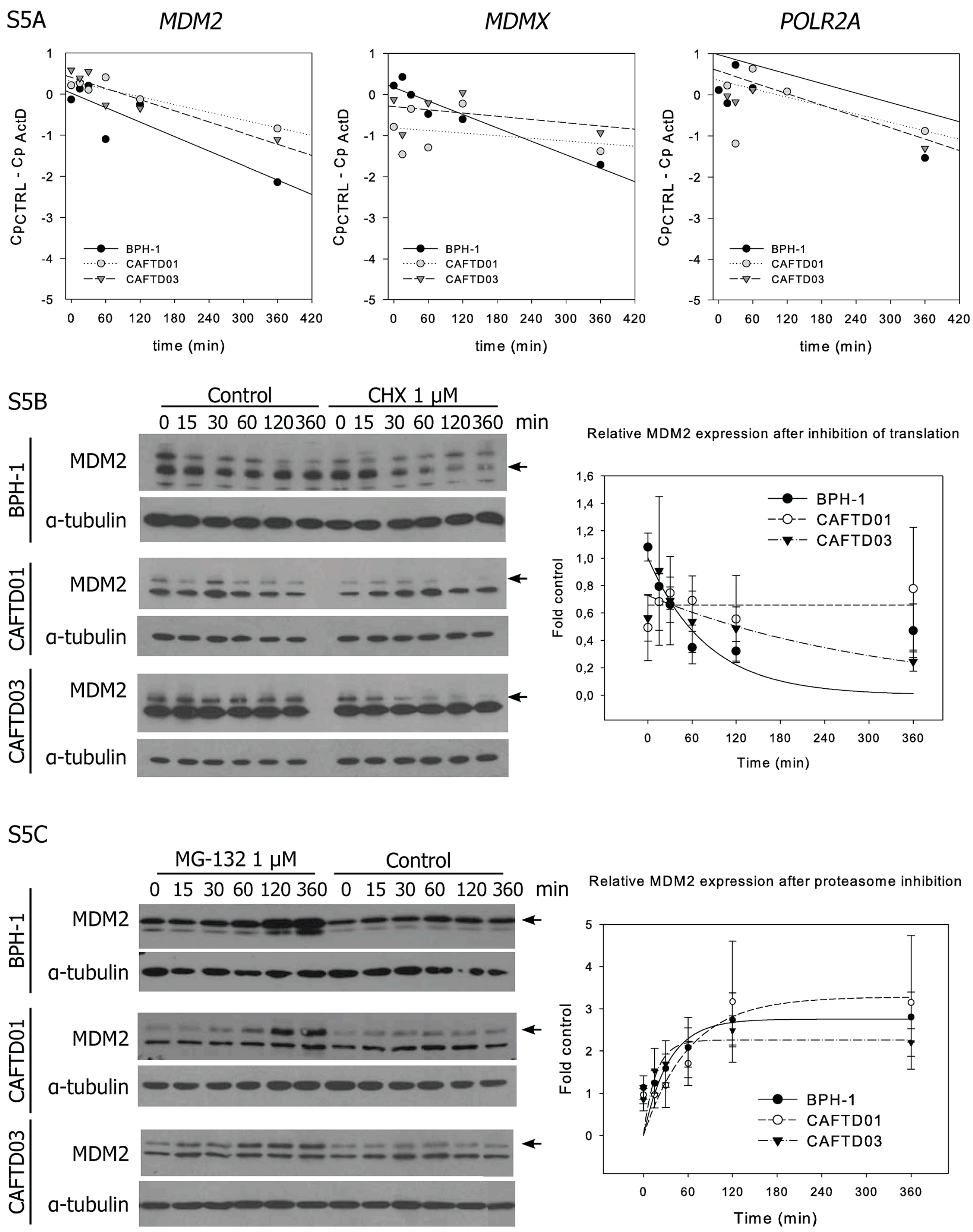


**Supplementary Figure S5: Downregulation of MDM2 in CAFTD clones is not a result of altered mRNA or protein stability. S5A.** For analysis of mRNA stability, the cells were treated with 0.3125 µM actinomycin D (ActD) and RNA was isolated at the indicated time points, reverse-transcribed, and analyzed by qRT-PCR. The graphs represent differences in Cp values between ActD-treated cells and untreated controls. **S5B, S5C.** For analysis of protein stability, MDM2 expression was evaluated after treatment with 1 µM cycloheximide (CHX) or 1 µM MG-132 for the indicated time intervals. The graphs show the mean ± SD of OD-based protein quantifications from three independent experiments.

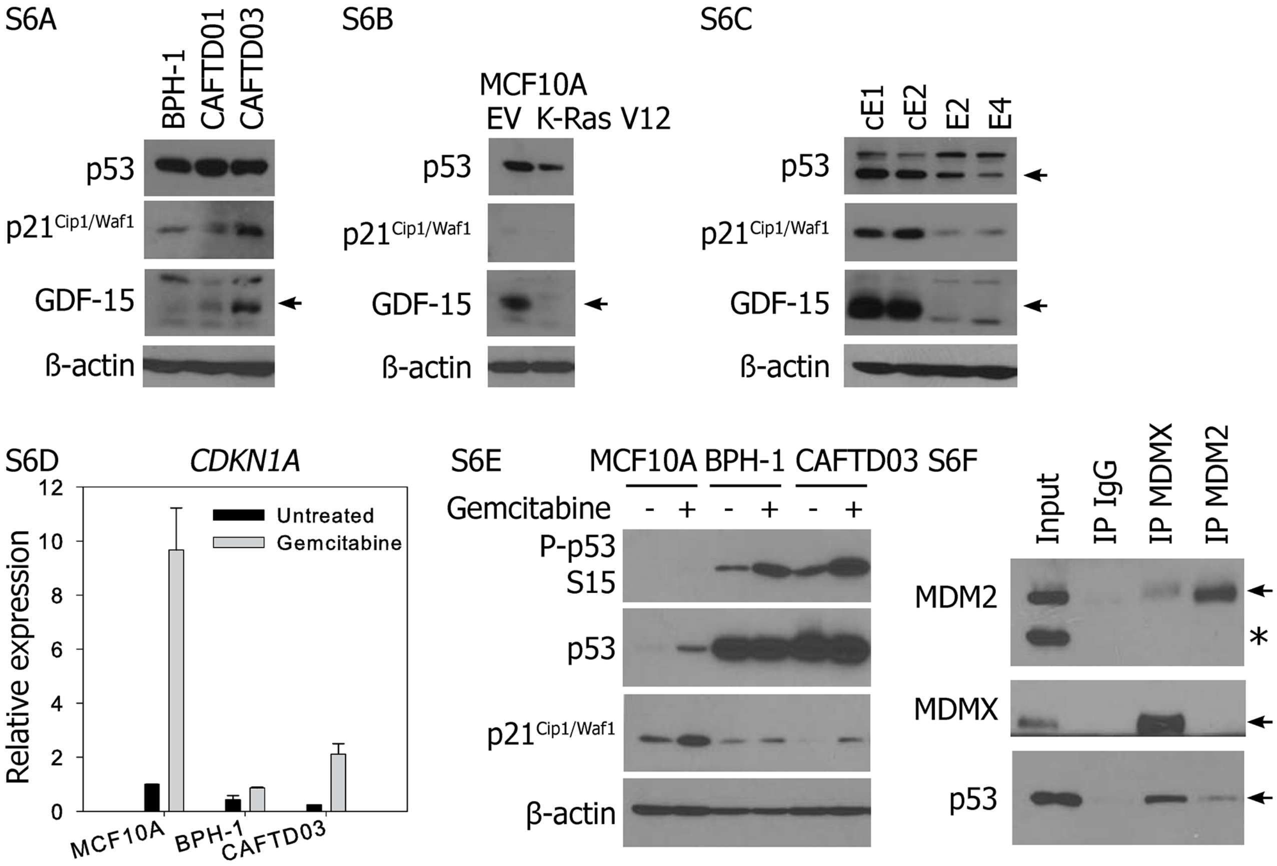


**Supplementary Figure S6: Analysis of p53 expression and its downstream targets in the presence or absence of DNA damage. S6A–S6C.** Analysis of p53 expression and its downstream targets p21$^{Cip1/Waf1}$ and GDF-15 by western blotting in untreated cells. Massive p53 expression in BPH-1 and both CAFTD clones results from SV40 immortalization, increased expression of both p53 targets is observed in CAFTD03 cells. The specific GDF-15 and murine p53 product is marked by an arrow. **S6D–S6E.** BPH-1 and CAFTD cells exhibit defects in DNA damage-induced p53 response. The indicated cells were treated with 16.7 nM gemcitabine for 24 h, followed by qRT-PCR analysis of a p53 target, *CDKN1A*, and western blot analysis of molecules implicated in the DNA damage-signaling pathway (phosphorylated p53 [P-p53 S15]) and induction of p21$^{Cip1/Waf1}$. qRT-PCR analysis of *CDKN1A* shows mean ± SEM of three independent experiments. **S6F.** Physical interaction of MDM2, MDMX, and p53 is not impaired in SV40-immortalized cells. MDM2 and MDMX were immunoprecipitated from untreated CAFTD03 cells and the indicated proteins were detected by western blotting.

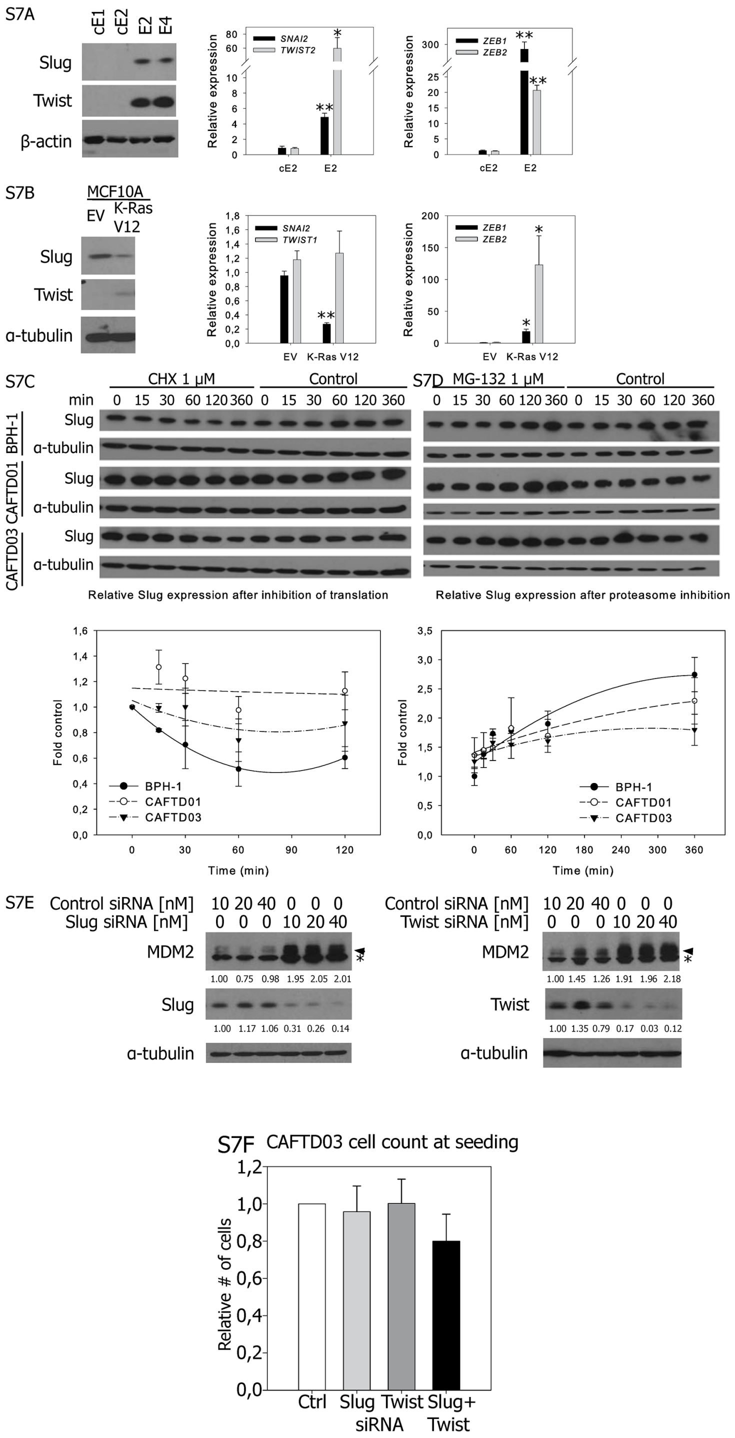


**Supplementary Figure S7: EMT of prostate and breast cell lines is accompanied by upregulation of EMT-driving transcription factors and stability of Slug protein in BPH-1 and CAFTD cells. S7A–S7B.** Western blotting and qRT-PCR analysis of EMT-driving transcription factors. PCR data represent mean ± SEM of at least three independent experiments. *$P < 0.05$; **$P < 0.01$. **S7C–S7D.** Stability of Slug protein expression was evaluated after treatment with 1 µM CHX or 1 µM MG-132 for the indicated time intervals. The graphs show mean ± SD values of OD-based protein quantifications from three independent experiments. **S7E.** CAFTD03 cells were transfected with three different concentrations of Slug- or Twist-specific siRNA, followed by western blotting analysis of MDM2 expression 48 h after transfection. The full-length MDM2 protein product is marked by an arrow; a faster-migrating product observed in human cells is marked by an asterisk. **S7F.** Downregulation of Slug and Twist does not significantly influence cell proliferation. Transfected cells were cultivated for 24 hours, serum-starved overnight and viable cells were counted prior seeding into Transwell chambers. The graphs show average cell number from 3 independent experiments, $P > 5.10^{-2}$.

**Supplementary Table S1: Spearman's correlation coefficient R of MDM2, MDMX and selected EMT markers in clinical samples from CaP and its disseminations into LN and SV (*n* = 154) or BrCa and LN metastases (*n* = 48)**

| CaP | E-cadherin | Vimentin epithelial | p53 | MDM2 nucleus | MDM2 cytosol | MDMX nucleus | MDMX cytosol |
|---|---|---|---|---|---|---|---|
| E-cadherin | 1,0000 | −0,0043 | 0,0681 | −0,1459 | −0,0044 | −0,0103 | −0,0311 |
| Vimentin epithelial | −0,0043 | 1,0000 | 0,0727 | −0,0057 | 0,0067 | **0,1699** | 0,1140 |
| p53 | 0,0681 | 0,0727 | 1,0000 | **0,2311** | −0,1093 | **0,2992** | 0,0289 |
| MDM2 nucleus | −0,1459 | −0,0057 | **0,2311** | 1,0000 | −0,0502 | 0,1176 | 0,0871 |
| MDM2 cytosol | −0,0044 | 0,0067 | −0,1093 | −0,0502 | 1,0000 | **−0,1987** | 0,0894 |
| MDMX nucleus | −0,0103 | **0,1699** | **0,2992** | 0,1176 | **−0,1987** | 1,0000 | **0,2382** |
| MDMX cytosol | −0,0311 | 0,1140 | 0,0289 | 0,0871 | 0,0894 | **0,2382** | 1,0000 |
| **BrCa** | **E-cadherin** | **Vimentin epithelial** | **p53** | **MDM2 nucleus** | **MDM2 cytosol** | **MDMX nucleus** | **MDMX cytosol** |
| E-cadherin | 1,0000 | −0,0684 | 0,0658 | 0,1792 | −0,0054 | −0,0095 | 0,1173 |
| Vimentin epithelial | −0,0684 | 1,0000 | **0,3987** | 0,2504 | 0,1504 | **0,2987** | 0,1647 |
| p53 | 0,0658 | **0,3987** | 1,0000 | 0,1809 | 0,1099 | 0,1824 | 0,2256 |
| MDM2 nucleus | 0,1792 | 0,2504 | 0,1809 | 1,0000 | 0,2350 | 0,1601 | 0,2101 |
| MDM2 cytosol | −0,0054 | 0,1504 | 0,1099 | 0,2350 | 1,0000 | 0,2889 | 0,0994 |
| MDMX nucleus | −0,0095 | **0,2987** | 0,1824 | 0,1601 | 0,2889 | 1,0000 | 0,0507 |
| MDMX cytosol | 0,1173 | 0,1647 | 0,2256 | 0,2101 | 0,0994 | 0,0507 | 1,0000 |

For highlighted scores, $P < 0.05$

**Supplementary Table S2: Antibodies for western blotting**

| Protein | Source | Catalog No. | Dilution |
|---|---|---|---|
| Akt 1,2,3 | Santa Cruz Biotechnology | sc-8312 | 1:1 000 |
| E-cadherin | BD Pharmingen | 610182 | 1:2 000 |
| ERK | Cell Signaling | #9102 | 1:1 000 |
| GAPDH | Santa Cruz Biotechnology | sc-20357 | 1:500 |
| GDF-15 | Millipore | 07-217 | 1:250 |
| Id1 | Santa Cruz Biotechnology | sc-488 | 1:500 |
| Lamin B | Santa Cruz Biotechnology | sc-6217 | 1:500 |
| MDM2 2A10 | B. Vojtěšek | | 1:2 |
| MDM2 2A9 | B. Vojtěšek | | 1 μg/ml |
| MDMX | Millipore | 04-1555 | 1:1 000 |
| MDMX | Abnova | H00004194-D01 | 1:1 000 |
| N-cadherin | BD Pharmingen | 610920 | 1:2 000 |
| p21 | Cell Signaling | #2947 | 1:1 000 |
| p53 | Santa Cruz Biotechnology | sc-126 | 1:1 000 |

(*Continued*)

| Protein | Source | Catalog No. | Dilution |
|---|---|---|---|
| P-Akt S473 | Cell Signaling | #9271 | 1:1 000 |
| P-ERK T202/Y204 | Cell Signaling | #9101 | 1:1 000 |
| P-H2A.X | Cell Signaling | #9718 | 1:500 |
| P-p53 S15 | Cell Signaling | #9286 | 1:1 000 |
| P-Smad2 S465/467 | Cell Signaling | #3101 | 1:1 000 |
| Slug | Cell Signaling | #9585 | 1:1 000 |
| Smad 2/3 | Cell Signaling | #3102 | 1:1 000 |
| Twist | Santa Cruz Biotechnology | sc-81417 | 1:300 |
| Vimentin | Sigma-Aldrich | V6389 | 1:500 |
| Vimentin | Sigma-Aldrich | SAB4300676 | 1:500 |
| α-tubulin | Sigma-Aldrich | T9026 | 1:4 000 |
| β-actin | Sigma-Aldrich | A5441 | 1:4 000 |
| anti-mouse IgG-HRP | GE Healthcare Biosciences | NA931 | 1:4 000 |
| anti-rabbit IgG-HRP | GE Healthcare Biosciences | NA934 | 1:3 000 |
| anti-goat IgG-HRP | Sigma-Aldrich | A4174 | 1:8 000 |
| **Antibodies for immunohistochemistry** | | | |
| β-catenin | Santa Cruz | sc-7963 | 1:20 |
| E-cadherin | Dako | IR059 | 1:50 |
| MDM2 | B. Vojtěšek | 2A9 | 1:50 |
| MDMX | Bethyl Laboratories | IHC-00108 | 1:75 |
| MDMX | Sigma-Aldrich | HPA018919 | 1:100 |
| Slug | Cell signaling | #9585 | 1:100 |
| Vimentin | Dako | IR630 | Ready-to-use |
| Secondary antibody | Dako | Dual Link | Ready-to-use |
| **Antibodies for immunoprecipitation** | | | |
| MDM2 | B. Vojtěšek | 2A9 | |
| MDMX | Millipore | 04-1555 | |
| Unlabeled Mouse IgG | Southern Biotech | 0107-01 | |

**Supplementary Table S3: Sequences of primers used in quantitative RT-PCR with UPL library**

| Gene | Species | Accession No. | Oligonucleotide sequence | UPL probe | Assay efficiency |
|---|---|---|---|---|---|
| CDH1 | *Homo sapiens* | NM_004360.3 | F: CCCGGGACAACGTTTATTACR | #35 | 1.883 |
| Cadherin 1, type 1, E-cadherin (epithelial) | | | R: GCTGGCTCAAGTCAAAGTCC | | |
| CDKN1A | *Homo sapiens* | NM_000389.4 | F: CCGAAGTCAGTTCCTTGTGG | #82 | 2.004 |
| Cyclin-dependent kinase inhibitor 1A (p21, Cip1) | | | R: CATGGGTTCTGACGGACAT | | |
| ID1 | *Homo sapiens* | NM_002165.2 | F: CCAGAACCGCAAGGTGAG | #39 | 1.810 |
| Inhibitor of DNA binding 1 | | | R: GGTCCCTGATGTAGTCGATGA | | |
| MDM2[1] | *Homo sapiens* | NM_002392.3 | F: CCATGATCTACAGGAACTTGGTAGTA | #18 | 1.878 |
| MDM2 oncogene, E3 ubiquitin protein ligase | | | R: TCACTCACAGATGTACCTGAGTCC | | |
| MDM2[2] | *Homo sapiens* | NM_002392.3 | F: GACTCCAAGCGCGAAAAC | #68 | 2.295 |
| MDM2 oncogene, E3 ubiquitin protein ligase | | | R: GGTGGTTACAGCACCATCAGT | | |
| POLR2A | *Homo sapiens* | NM_000937.3 | F: GCAAATTCACCAAGAGAGACG | #1 | 2.062 |
| DNA-directed RNA polymerase II | | | R: CACGTCGACAGGAACATCAG | | |
| SNAI2 | *Homo sapiens* | NM_003068.3 | F: TGGTTGCTTCAAGGACACAT | #7 | 2.279 |
| Homo sapiens snail homolog 2 (Drosophila) | | | R: GTTGCAGTGAGGGCAAGAA | | |
| TWIST1 | *Homo sapiens* | NM_000474.3 | F: AGCTACGCCTTCTCGGTCT | #58 | 1.735 |
| Homo sapiens twist homolog 1 | | | R: CCTTCTCTGGAAACAATGACATC | | |
| VIM | *Homo sapiens* | NM_003380.2 | F: AAAGTGTGGCTGCCAAGAAC | #16 | 1.907 |
| Homo sapiens Vimentin | | | R: AGCCTCAGAGAGGTCAGCAA | | |
| ZEB1 | *Homo sapiens* | NM_001128128.1 | F: GCTGGGAGGATGACAGAAAG | #57 | 2.329 |
| Zinc finger E-box-binding homeobox 1 | | | R: TGCATCTGACTCGCATTCAT | | |
| ZEB2 | *Homo sapiens* | NM_014795.2 | F: AAGCCAGGGACAGATCAGC | #68 | 2.143 |
| Zinc finger E-box binding homeobox 2 | | | R: GCCACACTCTGTGCATTTGA | | |

[1]Targets exon boundary 5–6

[2]Targets exon boundary 1–2

## Sequences of primers used in quantitative RT-PCR with UPL library

| Gene | Species | Accession No. | Oligonucleotide sequence | UPL probe | Assay efficiency |
|---|---|---|---|---|---|
| CDH1 | *Mus musculus* | NM_009864.2 | F: ATCCTCGCCCTGCTGATT | #18 | 1.869 |
| Mus musculus cadherin 1 | | | R: ACCACCGTTCTCCTCCGTA | | |
| MDM2 | *Mus musculus* | NM_010786.3 | F: TGTTTGGAGTCCCGAGTTTC | #99 | 1.983 |
| Mus musculus transformed mouse 3T3 cell double minute 2 | | | R: AGCCACTAAATTTCTGTAGATCATTG | | |
| MDMX | *Mus musculus* | NM_008575.3 | F: GAAGTAGGGAAAACAAGTAACAAGAAG | #10 | 1.933 |
| Mdm4 p53 binding protein homolog (mouse) | | | R: TCAAGGACCTGGAGTCCTCA | | |
| TWIST2 | *Mus musculus* | NM_007855.2 | F: CATGTCCGCCTCCCACTA | #10 | 1.926 |
| Mus musculus twist homolog 2 | | | R: GATGTGCAGGTGGGTCCT | | |
| VIM | *Mus musculus* | NM_011701.3 | F: CCAACCTTTTCTTCCCTGAA | #109 | 2.038 |
| Mus musculus Vimentin | | | R: TGAGTGGGTGTCAACCAGAG | | |
| ZEB1 | *Mus musculus* | ENSMUST00000025081.5 | F: AGGTGATCCAGCCAAACG | #93 | 1.873 |
| Zinc finger E-box-binding homeobox 1 | | | R: GGTGGCGTGGAGTCAGAG | | |
| ZEB2 | *Mus musculus* | ENSMUST00000076836.5 | F: AAGCCAGGGACAGATCAGC | #68 | 1.938 |
| Zinc finger E-box binding homeobox 2 | | | R: CACACTCCGTGCACTTGAA | | |

## Custom and inventoried assays used in quantitative RT-PCR

| Gene | Species | Accession No. | Assay ID | Provider |
|---|---|---|---|---|
| MDMX<br>Homo sapiens MDM4, p53 regulator | *Homo sapiens* | NM_002393.4 | RealTime ready Catalog assay Id 111132 | Roche |
| SNAI2<br>Mus musculus snail family zinc finger 2 | *Mus musculus* | NM_011415.2 | RealTime ready Catalog assay Id 311697 | Roche |
| GAPD<br>Mus musculus glyceraldehyde-3-phosphate dehydrogenase | *Mus musculus* | NM_008084 | Cat. no. 05046211001 | Roche |
